# Bilateral Angular Gyrus 810-nm Transcranial Near-Infrared Stimulation Reshapes Brain Network: Ameliorate Alzheimer’s Disease Cognitive Deficits

**DOI:** 10.34133/bmef.0313

**Published:** 2026-09-22

**Authors:** Xiaowei Ma, Fangyuan Yan, Zhanxu Liu, Sha He, Li Liu, Jiayi Liang, Mengwei Yuan, Shuaixiang Wang, Shuo Qu, Songqi Yang, Yan Gao, Huiqing Qiu, Yang Li, Chris Yang, Yiming Li, Ting Li, Yuping Wang

**Affiliations:** ^1^ Department of Neurology, The First Hospital of Hebei Medical University, Shijiazhuang, China.; ^2^ Department of Neurology, Hebei Hospital of Xuanwu Hospital Capital Medical University, Shijiazhuang, China.; ^3^ Neuromedical Technology Innovation Center of Hebei Province, Shijiazhuang, China.; ^4^ Department of Graduate School, Hebei Medical University, Shijiazhuang, China.; ^5^ Institute of Biomedical Engineering, Chinese Academy of Medical Sciences & Peking Union Medical College, Tianjin, China.; ^6^ School of Engineering, Cardiff University, Cardiff, UK.; ^7^ Department of Engineering, University of Cambridge, Cambridge, UK.; ^8^ Institute of Intelligent Medicine, Chinese Academy of Medical Sciences & Peking Union Medical College, Beijing, China.; ^9^ Department of Neurology, Xuanwu Hospital, Capital Medical University, Beijing, China.; ^10^ Beijing Key Laboratory of Neuromodulation, Beijing, China.; ^11^ Center for Sleep and Consciousness Disorders, Beijing Institute for Brain Disorders, Beijing, China.; ^12^ Center of Epilepsy, Beijing Institute for Brain Disorders, Capital Medical University, Beijing, China.; ^13^ Collaborative Innovation Center for Brain Disorders, Capital Medical University, Beijing, China.

## Abstract

**Objective:** To investigate the clinical efficacy and neural mechanisms of targeted 810-nm transcranial near-infrared stimulation (tNIRS) over the bilateral angular gyrus in Alzheimer’s disease (AD). **Impact Statement:** We provide the first clinical evidence that targeted tNIRS ameliorates AD cognitive deficits by dynamically reconfiguring large-scale brain networks, establishing a precise, mechanistic neuromodulation strategy. **Introduction:** While tNIRS shows potential for AD, conventional whole-brain irradiation lacks anatomical precision and yields inconsistent outcomes. Resolving these elusive network-level mechanisms requires targeted stimulation paradigms. **Methods:** In a randomized, sham-controlled trial, 36 biomarker-confirmed AD patients received 810-nm tNIRS targeted at the bilateral angular gyrus or sham stimulation (20 min/side daily, 15 d). Baseline and post-intervention neuropsychological assessments, resting-state functional magnetic resonance imaging (fMRI), and transcranial magnetic stimulation–electroencephalography (TMS–EEG) were utilized to decode cognitive improvements and network dynamics. **Results:** Targeted tNIRS obviously improved cognition, memory, language, attention, and executive functions while reducing neuropsychiatric symptoms. fMRI revealed enhanced intra-network connectivity within the default mode network (DMN) and promoted functional synergy between the DMN, dorsal attention, frontoparietal, and limbic networks. TMS–EEG demonstrated enhanced information flow and dynamic nodal reconfiguration, specifically boosting prefrontal and temporo-occipital activation. **Conclusion:** Bilateral angular gyrus targeted tNIRS may effectively mitigate AD cognitive impairment potentially through modulation of functional connectivity and adaptively reconfiguring higher-order cognitive networks, serving as a highly promising clinical neuromodulation therapy.

## Introduction

As global population aging intensifies, the incidence of Alzheimer’s disease (AD) is increasing annually [1,2]. Conventional, drug-based treatment for AD has many limitations, such as side effects, high costs, limited efficacy, and challenges in research and development [3,4]. Therefore, the need for novel and effective interventions to combat AD is pressing.

Recent studies have demonstrated that transcranial near-infrared stimulation (tNIRS) can enhance cognitive function in patients with AD [5,6]. tNIRS employs near-infrared light, typically in the 600- to 1,100-nm wavelength range, which exhibits unique optical attributes such as deep tissue penetration, low absorption by water and hemoglobin, and high scattering within biological tissues [7,8]. These optical attributes of tNIRS empower neurologists to employ light as a natural transmission medium, effectively traversing the dermal and cranial barriers to establish direct interaction with the cerebral parenchyma [8,9]. Through this direct engagement, tNIRS activates biochemical processes that sustain a healthy cerebral environment, thereby providing an effective intervention strategy for both acute and chronic pathological brain conditions [7,10–12]. Rodent studies have confirmed that this technology can enhance adenosine triphosphate synthesis by activating mitochondrial cytochrome oxidase, stimulate vascular endothelial growth factor secretion, improve metabolic waste clearance through meningeal lymphatic vessels, and reduce oxidative stress, inflammatory responses, tau protein hyperphosphorylation, and amyloid-β (Aβ) plaque deposition, thus providing a theoretical foundation for its use to ameliorate the pathological microenvironment in AD [13–21]. Controlled experiments in healthy young and older adult cohorts have demonstrated that tNIRS modulates key neurobiological processes, including neurosynaptic plasticity, enhances resting-state functional connectivity (FC), and optimizes cerebral oxygenation dynamics. These mechanistic improvements reportedly correlate with marked enhancements in working memory performance and executive function metrics among intervention groups [22–24].

Clinical reports on tNIRS therapy for dementia have consistently demonstrated excellent safety profiles, and no severe adverse events have been reported. Improvements in participants’ cognitive function, emotional state, and health-related quality of life have also been reported. The therapeutic benefits appear to be primarily achieved through whole-brain multifocal stimulation, with treatment protocols lasting 8 to 12 weeks [25–28]. Current tNIRS protocols predominantly rely on a nonspecific, whole-brain irradiation approach. The lack of precise anatomical targeting contributes to inconsistent therapeutic outcomes. Since AD is increasingly understood as a disorder of brain network dysregulation, there is a compelling need for randomized controlled trials that employ precise, target-specific stimulation. Such studies are essential to establish the efficacy of modulating key neural hubs and to elucidate the underlying brain network mechanisms.

Aβ deposition in the brain of patients with AD reportedly first appears in the posterior cingulate gyrus, prefrontal cortex, anterior cuneus, and temporoparietal regions, and the reduction of FC in these regions reportedly affects the functional integrity of the default mode network (DMN) [29–31]. As a core hub of the DMN and a potential convergence zone in AD, the angular gyrus (AG) is involved in memory retrieval and formation [32]. Furthermore, gray matter volume and functional coupling within both AG are also associated with cognitive ability in patients with AD. Additionally, the AG exhibits extensive structural connectivity and FC with other brain regions, including the medial prefrontal cortex, temporal cortex, precuneus, and superior and inferior frontal gyri, playing a pivotal role in functional integration and participating in processes such as perceptual attention and decision-making [33,34]. Previous experiments involving noninvasive neuromodulation treatment for AD that targeted the AGs demonstrated improvements in patients’ cognitive function and amelioration of their neuropsychiatric symptoms; they also revealed important increases in FC within the DMN regions, along with enhanced local, long-range, and dynamic connectivity across the brain [35,36]. Therefore, tNIRS that targets both AG may ameliorate declines in the function of this core hub and facilitate information integration across different cognitive domains, thereby constituting an effective AD therapy.

The brain network is a focal point in AD research, and our understanding of the pathogenesis of AD is shifting toward a systemic framework of large-scale brain network dysfunction [37,38]. During the progression of AD, the DMN is especially vulnerable owing to its high metabolic demands and synaptic complexity. Dysfunctional integration among higher-order cognitive networks, including the DMN, dorsal attention network (DAN), frontoparietal network (FPN), and limbic network (LN), also leads to dissociation between memory encoding and executive function [39–42]. The global efficiency of whole-brain networks progressively diminishes because of the loss of white matter tract integrity, synaptic loss in hub nodes, and weakened neural synchrony [43,44]. Functional magnetic resonance imaging (fMRI) is a critical, noninvasive neuroimaging modality for the mapping of alterations in brain activity patterns and FC, facilitating mechanistic investigations into the effects of neuromodulatory interventions. Complementary to this technique, the integration of transcranial magnetic stimulation (TMS) with electroencephalography (EEG) uniquely enables real-time investigation of dynamic brain network interactions, offering precise quantification of neural information propagation dynamics with millisecond-level temporal resolution [45,46]. Therefore, the integration of fMRI with TMS–EEG enables the more comprehensive study of FC and information transfer within the brain of patients with AD.

To address some of the abovementioned knowledge gaps in the field, we conducted a randomized, sham-controlled, blinded trial. We aimed to investigate whether 810-nm tNIRS that targets the AG can improve cognitive function in patients with AD, as this technique is both portable and safe. We employed neuropsychological assessments to evaluate the efficacy of tNIRS applied over a clinically relevant 15-d period in patients with mild AD. Additionally, fMRI and TMS–EEG data were collected from the active stimulation group. Pre-stimulation versus post-stimulation changes in resting-state FC, whole brain network nodal properties, connectivity strength, and efficiency were analyzed to help elucidate the intrinsic neuromodulatory mechanisms. We hypothesized that tNIRS would enhance cognitive function in patients with AD by optimizing resting-state functional brain networks and time-varying network dynamics. With this study, we sought to establish a novel paradigm for tNIRS therapy in AD research.

## Results

### Overview of tNIRS treatment paradigm

**Fig. 1.**
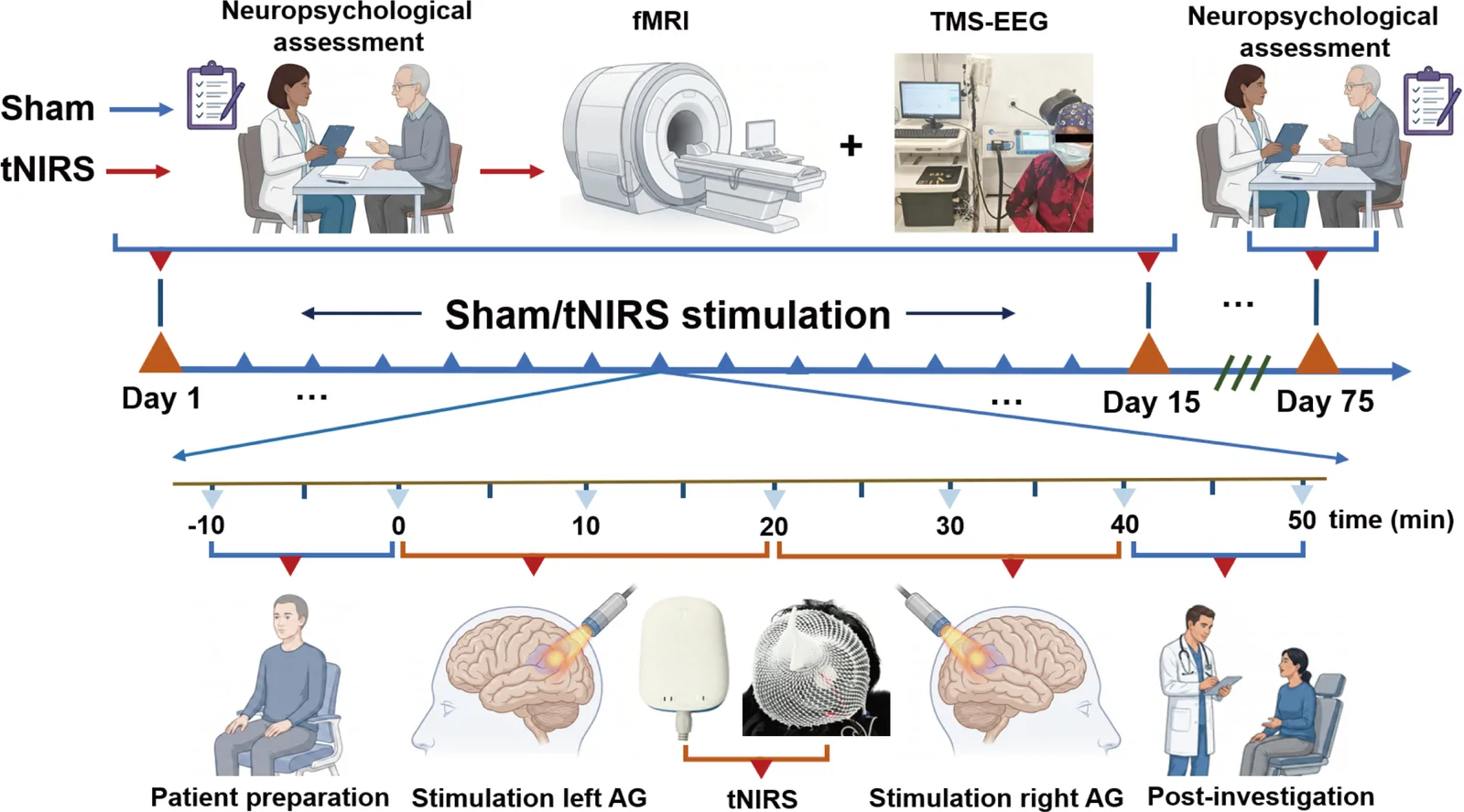
Experiment flow chart. Participants were divided into a phototherapy group and a sham stimulation group, both undergoing 15 d of intervention. Preparation occurred 10 min before each intervention, and safety assessment was performed within 10 min of the intervention. tNIRS group—Day 1: Underwent fMRI, TMS–EEG, and neuropsychological assessments, followed by phototherapy initiation. Days 2 to 14: Received only phototherapy. Day 15: After phototherapy, underwent fMRI, TMS–EEG, and neuropsychological assessments. Day 75: Underwent only neuropsychological assessments. Sham group—Day 1: Underwent neuropsychological assessments followed by sham stimulation. Days 2 to 14: Received only sham stimulation. Day 15: After sham stimulation, underwent neuropsychological assessments. After each session, study personnel checked for adverse reactions and documented them.

To systematically investigate the clinical efficacy and underlying neural mechanisms of bilateral AG-targeted tNIRS in AD, we designed a randomized, sham-controlled, and blinded clinical trial (Fig. 1). A total of 36 patients with mild AD were enrolled and randomly assigned to either the active tNIRS group (*n* = 18) or the sham stimulation group (*n* = 18). To comprehensively evaluate the multidimensional effects of the intervention, neuropsychological assessments, resting-state fMRI, and TMS–EEG were conducted at specific time points across a 75-d period.

Prior to analyzing the clinical outcomes and network dynamics, we established the specific tNIRS intervention paradigm and validated its optical feasibility. The bilateral AG target regions were determined by the 10-20 International EEG system, corresponding to the P3/P4 positions of the scalp. All patients were asked to cut their hair short at both sites to expose the scalp. In the tNIRS group, participants received 20 min of light exposure over the bilateral AG for 15 consecutive days. For the selection of wavelength, we referred to the previous study by Zhang et al. [47]. They conducted transcranial photobiomodulation (tPBM) optical simulations across 4 representative brain regions (the prefrontal, parietal, occipital, and temporal lobes) at 4 wavelengths (660, 810, 980, and 1,064 nm). The results demonstrated that in the temporal lobe, which is adjacent to the AG, the photon fluence attenuation curves for 660, 980, and 1,064 nm nearly overlapped. In contrast, the 810-nm wavelength maintained a significant advantage in photon transmission, with its photon fluence remaining above the effective threshold at a depth of approximately 4.5 cm. A core prerequisite for treating AD is that a sufficient dose of photons must penetrate the skull and reach the deep brain parenchyma to trigger a photobiomodulation response. Regarding the critical metric of photon delivery to the brain parenchyma, the 810-nm wavelength is obviously superior to 660 nm. Furthermore, compared to 980 and 1,064 nm, 810 nm exhibits a lower absorbed fraction and a higher transmission fraction. Consequently, this ensures that sufficient optical energy can precisely reach the pathological areas during tPBM treatment for AD.

The active stimulation group received 810-nm near-infrared light stimulation at an intensity of 85 mW/cm^2^ and a stimulation area of 0.8 cm^2^ (a circular area of 1 cm in diameter). In the sham group, an identical-appearing spacer without light emission was placed on the corresponding scalp locations (P3/P4). The sham device produced no light, heat, vibration, or sound, and was indistinguishable in appearance from the active device. Both active and sham groups received the same treatment duration (20 min per side, daily for 15 consecutive days), the same device placement procedure, and identical operator interactions. All participants were instructed to remain seated comfortably with their eyes closed during stimulation. Importantly, 810-nm tNIRS produces no perceptible heat, vibration, or sound at the scalp surface, making effective blinding feasible. Both near-infrared light stimulation and sham stimulation were provided by a transcranial photoelectric synchronous neuromodulator (Hunan Secrek Medical Technology Co. Ltd., Hunan, China), and the subjects sat comfortably in a recliner to receive stimulation.

### Bilateral AG region optical parameter analysis based on optical Monte Carlo simulation

To accurately evaluate the photon fluence distribution of 810-nm tPBM in the bilateral AG region of the human brain, we employed the Monte Carlo model of photon migration in voxelized media (MCVM) [47,48] to construct an optical simulation framework based on a high-resolution human anatomical model. This simulation was designed to verify whether photons can effectively penetrate brain tissues to ultimately and precisely deliver optical energy to the bilateral AG.

**Fig. 2.**
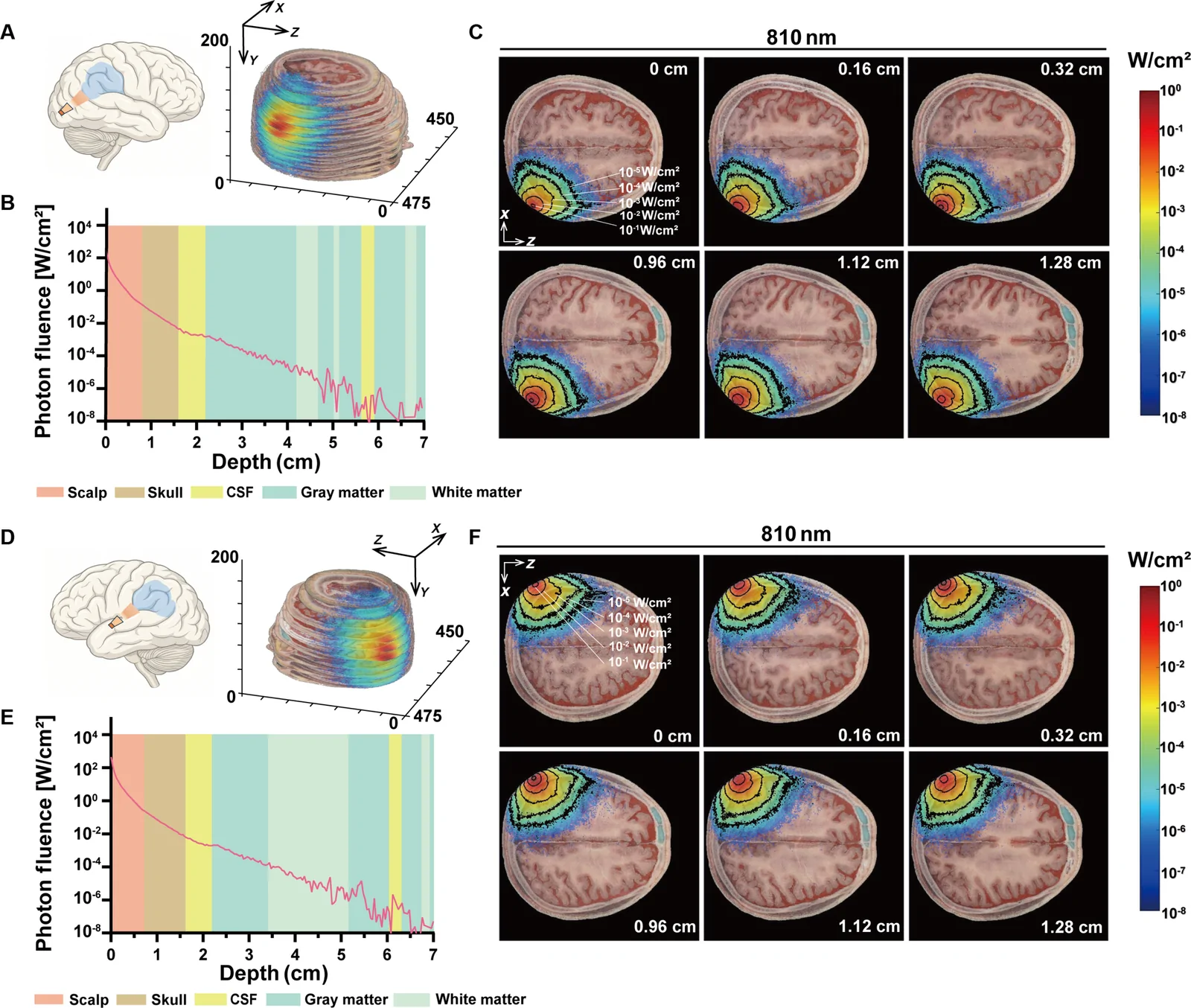
Photon fluence distribution in the bilateral AG based on MCVM. (A) Light propagation in the left AG region. (B) Photon fluence changes with the depth of penetration in the left AG region. (C) Photon fluence distribution of different slices in the left AG region. (D) Light propagation in the right AG region. (E) Photon fluence changes with the depth of penetration in the right AG region. (F) Photon fluence distribution of different slices in the right AG region.

The high-precision 3-dimensional (3D) human head model utilized in this study was derived from the Visible Chinese Human (VCH) [49,50] dataset. The VCH dataset is based on a Chinese male anatomical specimen, whose entire body was transversely sectioned and imaged at intervals of 0.02 cm. The images within this dataset were meticulously annotated by professional anatomists. In this study, we performed 3D resampling on the VCH head dataset to construct a 200 × 475 × 450 voxel matrix (Fig. 2A and D) with a resolution of 0.04 × 0.04 × 0.04 cm^3^. Based on the cerebral anatomical structure, the simulation model was precisely segmented into multiple tissue types with distinct optical properties, including the scalp, skull, cerebrospinal fluid (CSF), gray matter, and white matter. At a wavelength of 810 nm, each tissue type was assigned a specific absorption coefficient, scattering coefficient, and anisotropy factor, thereby ensuring the biophysical realism of the photon propagation simulation. To ensure methodological consistency and comparability with previous MCVM-based tPBM studies, all simulation parameters and model assumptions were adopted from the MCVM-based studies by Zhang et al. [47] and Yang et al. [48]. Specifically, the VCH human head model, tissue segmentation strategy, wavelength-dependent optical properties, photon-transport rules, boundary condition handling, source normalization, and fluence-calculation procedures followed the established MCVM framework described in those studies. The present analysis represents an AG-focused application of the previously validated MCVM simulation protocol, with the light source position adapted to the bilateral AG region according to the stimulation paradigm used in this clinical trial.

The AG of the human brain is located above Wernicke’s area, at the junction of the parietal and occipital lobes. In biological tissues, the attenuation of photon fluence not only depends on depth but also is profoundly influenced by the hierarchical structure of the tissue. As illustrated in Fig. 2A and D, MCVM simulation results visually reveal the photon propagation dynamics of 810-nm near-infrared light within this specific region. For the incident light source, the Gaussian source with a 1-cm diameter was applied to both the left and right AG.

Penetration depth is a critical determinant of the clinical efficacy of tPBM. By extracting the attenuation curves of photon fluence as a function of depth along the incident optical axis (the *x* axis) (Fig. 2B for the left area and Fig. 2E for the right AG), a rapid exponential decay in energy is clearly observed as photons traverse the scalp and skull. Once the photons penetrate the cranial barrier, the attenuation slope of the photon fluence within the brain parenchyma flattens obviously, because the optical scattering properties of gray and white matter are higher than their absorption properties. This wavelength specific characteristic of low absorption and high scattering enables photons to generate a widespread scattering effect within the AG. Upon entering the gray and white matter regions, the local photon fluence is still maintained between 10^−3^ and 10^−8^ W/cm^−2^.

Furthermore, the 2D tomographic views (Fig. 2C and F) detail the spatial diffusion range of optical energy across the cross-section (*X*–*Z* plane). Across a series of slices at varying depths, the contour lines (white) clearly demonstrate a broad spatial diffusion pattern of photons within the brain tissue of the bilateral AG. Even at the peripheral slices, the AG remains covered by an effective optical field.

### Demographic and clinical characteristics

In this study, 36 patients diagnosed with AD via positron emission tomography–computed tomography or analysis of CSF at our research center were enrolled (18 per group), all of whom completed the 15-d intervention and 60-d follow-up periods for efficacy analysis. At baseline, no significant differences were observed in sex, age, educational attainment, AD duration, or Mini-Mental State Examination (MMSE), Montreal Cognitive Assessment (MoCA), or Clinical Dementia Rating (CDR) scores (Table 1).

**Table 1.** Demographic and clinical characteristics at baseline. Data are presented as (mean ± SD) or counts (%).

Variables	HC (*N* = 16)	Sham (*N* = 18)	tNIRS (*N* = 18)	*P* value
Gender (female), *n* (%)	10 (62.5%)	13 (72.2%)	11 (61.1%)	0.750 ^a^
Age (years)	68.5 ± 5.56	66.33 ± 8.05	69.44 ± 6.50	0.083 ^b^
Education (years)	10.81 ± 3.96	10.56 ± 3.26	10.72 ± 4.30	0.869 ^b^
Duration (years)	-	1.81 ± 1.06	2.25 ± 1.32	0.273 ^c^
MMSE	-	20.94 ± 2.80	20.83 ± 2.45	0.689 ^c^
MoCA	-	15.33 ± 3.45	16.33 ± 3.50	0.208 ^c^
CDR	-	0.5 (50%)	0.5 (50%)	1 ^a^

tNIRS, transcranial near-infrared light stimulation; HC, healthy controls; MMSE, Mini-Mental State Examination; MoCA, Montreal Cognitive Assessment; CDR, Clinical Dementia Rating

^a^ The *P* value was obtained by Fisher’s exact test.

^b^ The *P* value was obtained by Welch’s ANOVA test.

^c^ The *P* value was obtained using 2-sample *t* test.

### Neuropsychology analysis

In all neuropsychological assessments, post-treatment differences were observed between the active and sham stimulation groups (all time × group interactions, *P* < 0.05). Within-group comparisons revealed varying degrees of treatment efficacy in the true stimulation group, while no significant differences were observed in the sham group.

**Fig. 3.**
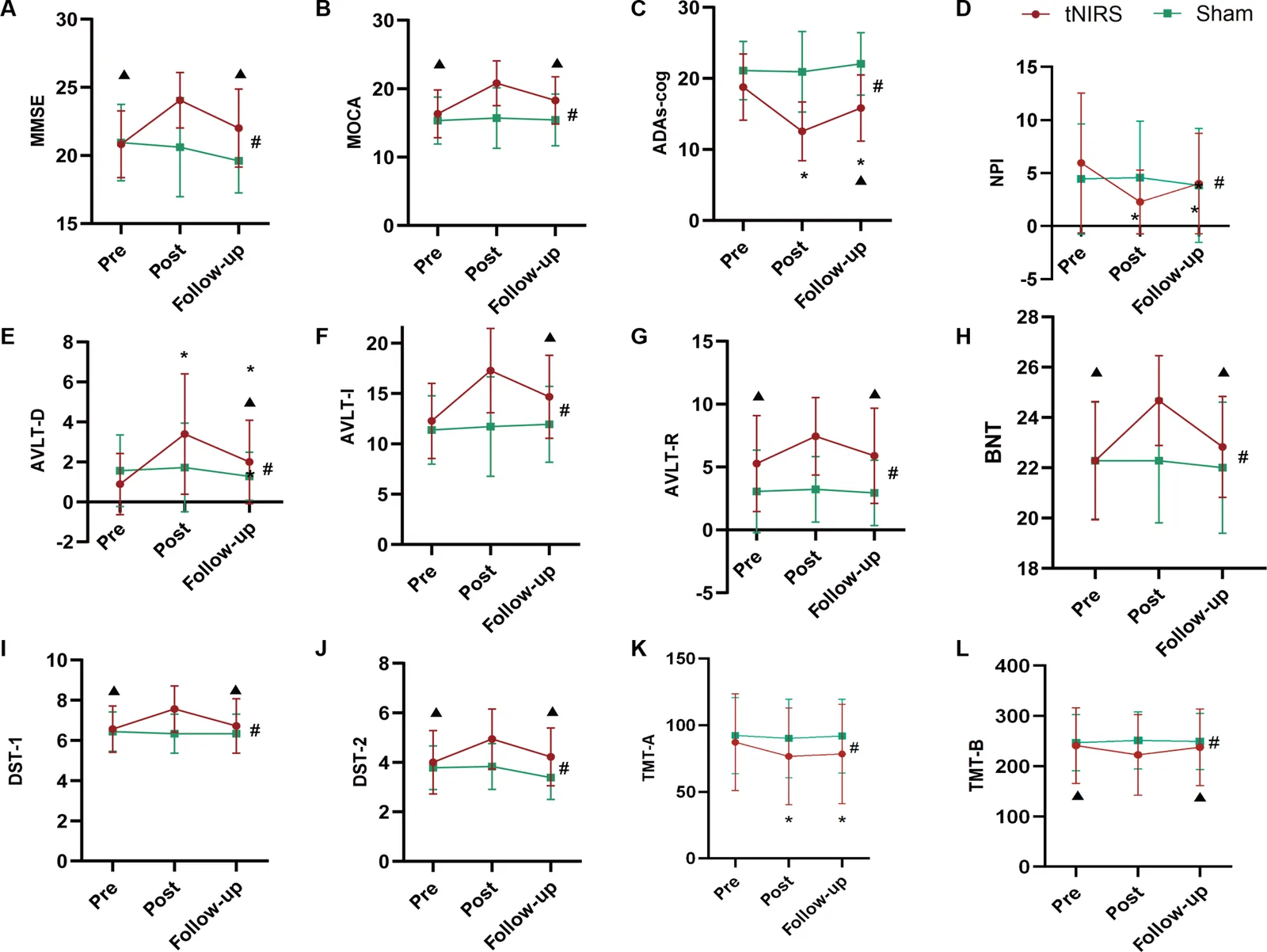
Results of the neuropsychological measurement. (A) Scores of Mini-Mental State Examination. (B) Scores of Montreal Cognitive Assessment. (C) Scores of Alzheimer’s disease assessment scale-cognitive section-cognitions. (D) Scores of Neuropsychiatric Inventory. (E) The scores of Rey Auditory Verbal Learning Test-Immediate Recall. (F) Scores of Rey Auditory Verbal Learning Test-Delayed Recall. (G) Scores of Rey Auditory Verbal Learning Test—Recognition. (H) Scores of Boston Naming Test. (I) Scores of Digit Span Test-1. (J) The scores of Digit Span Test-2. (K) The scores of Trail Making Test-A. (L) The scores of Trail Making Test-B. *: versus pre (day 0), *P* < 0.0167; ^▲^: versus post (day15), *P* < 0.0167; ^#^: Group × Time interaction, *P* < 0.05.

In the tNIRS group, improvements were observed in the 15-d post-treatment MMSE, MoCA, and ADAS-cog (Alzheimer’s Disease Assessment Scale-Cognitive Section) scores (Fig. 3A to C), compared with baseline (all *P* < 0.0167). The improvements in the MMSE and MoCA scores had weakened by day 60 (both *P* < 0.0167) and were no longer different from baseline (MMSE: *P* = 0.028; MoCA: *P* = 0.019). The therapy had a longer-lasting effect on the ADAS-cog score, with the improvement still evident by day 60 compared with baseline (*P* = 0.002), although it was lower than the immediate post-treatment effect (*P* = 0.006).

Improvements in the Neuropsychiatric Inventory (NPI) were observed by days 2 and 10 (Fig. 3D), compared with baseline (both *P* < 0.0167). In the Rey Auditory Verbal Learning Test (AVLT) subtests (Fig. 3E to G), improvements were observed after treatment (all *P* < 0.0167). Improvements in the AVLT-I and AVLT-D scores had weakened by day 60 (both *P* < 0.0167), although the scores were still better than those at baseline (both *P* < 0.0167). The AVLT-R score no longer differed from that at baseline by day 60 (*P* = 0.053). The Boston Naming Test (BNT) score (Fig. 3H) had improved by day 15 but had weakened by day 60 (*P* < 0.0167) to no longer differ from that at baseline (*P* = 0.02). The Digit Span Test (DST) (forward and backward) scores (Fig. 3I and J) had improved by day 15 (both *P* < 0.0167) but had weakened by day 60 (both *P* < 0.0167) to no longer differ from those at baseline (DST-1: *P* = 0.018; DST-2: *P* = 0.046). The Trail Making Test (TMT) section A score (Fig. 3K) was better immediately after treatment and on day 60 (both *P* < 0.0167) than that at baseline, with no difference between the immediate post-treatment time point and day 60 (*P* = 0.071). The TMT section B score (Fig. 3L) had improvement by day 15 (*P* < 0.0167) but had weakened by day 60 (*P* < 0.0167) to no longer differ from that at baseline (*P* = 0.084).

To evaluate clinical significance, we referenced minimal clinically important difference thresholds from previous literature. In our tNIRS group, mean MMSE improvement of 2.17 points and ADAS-cog improvement of 3.67 points exceeded the respective thresholds, indicating clinically meaningful benefits.

### Comparison between healthy controls and pre-stimulation patients

After gaussian random field (GRF) correction (voxel-level *P* < 0.001, cluster-level *P* < 0.05), the AD group exhibited obviously lower low-frequency fluctuation amplitude (ALFF) in several brain regions compared with the healthy controls (HC) group, including the left superior frontal gyrus, left middle frontal gyrus, right anterior cuneus, left middle cingulate gyrus, left posterior cingulate gyrus, and left precuneus.

**Fig. 4.**
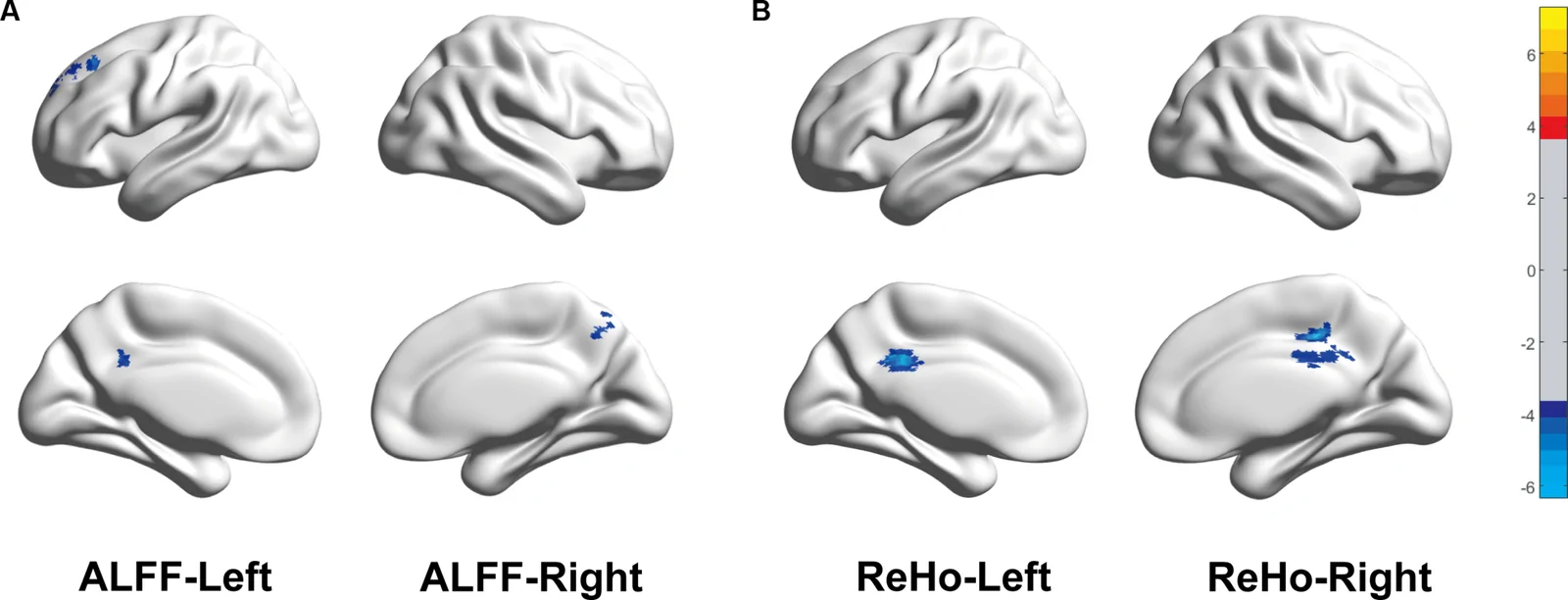
Regional differences in neurophysiology between healthy controls and pre-stimulation patients. (A) ALFF left and right hemisphere. (B) ReHo left and right hemisphere.

Consistently, following GRF correction (voxel-level *P* < 0.005, cluster-level *P* < 0.05), regional homogeneity (ReHo) was also significantly reduced in the AD group relative to HC. This reduction was localized within a widespread cluster in the posterior medial cortex, with its statistical peak in the left posterior cingulate gyrus. The cluster encompassed bilateral middle and posterior cingulate gyri and extended into the right precuneus (Fig. 4).

### Changes in function connectivity of fMRI

**Fig. 5.**
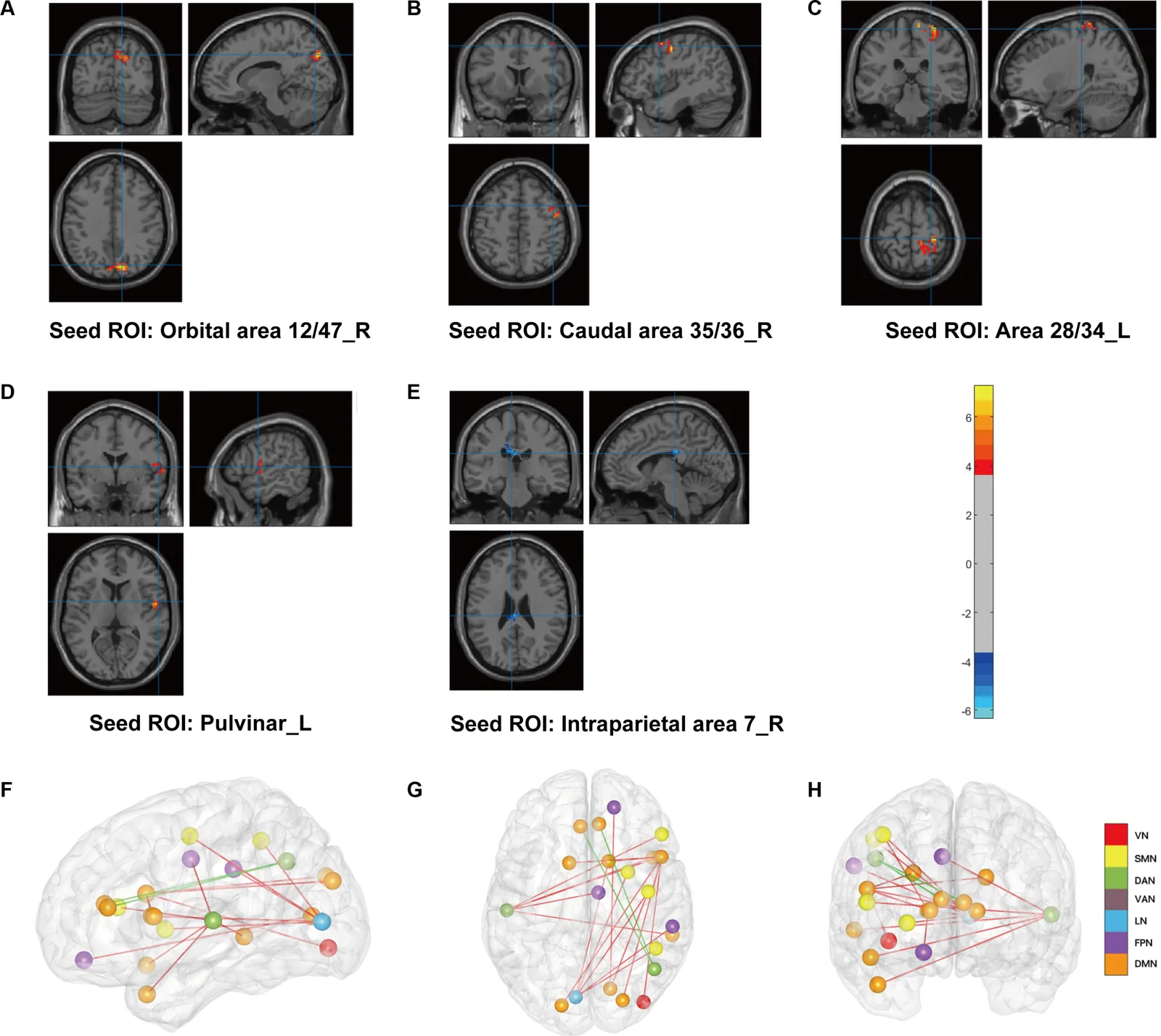
Resting-state functional connectivity of seed-ROI. (A) Brain cluster sFC changes with orbital area 12/47_R as the ROI after tNIRS. (B) Brain cluster sFC changes with Caudal area 35/36_R as the ROI after tNIRS. (C) Brain cluster sFC changes with Area 28/34_L (EC, entorhinal cortex) as the ROI after tNIRS. (D) Brain cluster sFC changes with a custom Pulvinar_L ROI after tNIRS. (E) Brain cluster sFC changes with the custom intraparietal area 7_R ROI after tNIRS. (F) Left-view FC visualization via BrainNet. (G) Top-view FC visualization via BrainNet. (H) Front-view FC visualization via BrainNet. VN, visual network; SMN, sensorimotor network; DAN, dorsal attention network; VAN, ventral attention network; LN, limbic network; FPN, frontoparietal network; DMN, default mode network. (A to E) Red cluster, enhanced region; blue cluster, decreased region. (F and G) Red edge, enhanced functional connectivity; green edge, decreased functional connectivity. Enhanced functional connectivity: DMN-DMN/VN/FPN/SMN/LN; LN-FPN/SMN/; DAN-SMN/FPN/DMN. Decreased functional connectivity: DAN-DMN.

Although neuropsychological scales have confirmed the significant clinical efficacy of 810-nm tNIRS at the macroscopic level, the underlying neural remodeling mechanisms remain a black box. To address this, we employed fMRI to investigate spatial connectivity changes in the brain networks of patients. In the selected regions of interest (ROIs), 5 seed regions exhibited significant post-stimulation changes in FC compared with the pre-stimulation baseline: the orbital area 12/47_R, caudal area 35/36_R, area 28/34_L [entorhinal cortex (EC)], pulvinar_L, and intraparietal area 7_R (Table 2 and Fig. 5A to E).

**Table 2.** Seed-based functional connectivity explanation

ROI	Peak MNI coordinate region	Network	Change
Orbital area 12/47_R (DMN)	Cuneus_R	DMN	+
	Cuneus_L	DMN	+
	Precuneus_R	DMN	+
	Precuneus_L	DMN	+
	Occipital_Sup_R	VN	+
Caudal area 35/36_R (DMN)	Frontal_Mid_2_R	FPN	+
	Precentral_R	SMN	+
Area 28/34_L (EC, entorhinal cortex) (LN)	Precentral_R	SMN	+
	Postcentral_R	SMN	+
	Paracentral_Lobule_R	SMN	+
	Parietal_Sup_R	FPN	+
	Precuneus_R	DMN	+
Pulvinar_L (DAN)	Rolandic_Oper_R	SMN	+
	Temporal_Sup_R	DMN	+
	Heschl_R	DMN	+
	Temporal_Pole_Sup_R	DMN	+
	Insula_R	FPN	+
Intraparietal area 7_R (DAN)	Cingulate Post L	DMN	−
	Cingulate Post R	DMN	−

DMN, default mode network; VN, visual network; SMN, sensorimotor network; LN, limbic network; FPN, frontoparietal network; DAN, dorsal attention network; +, enhanced functional connectivity; −, decreased functional connectivity

Among the 5 seed ROIs, 4 exhibited increased FC with other brain regions following stimulation. (a) Orbital area 12/47_R exhibited strengthened connectivity with both cunei, both precunei, and the right superior occipital cortex. (b) Caudal area 35/36_R exhibited enhanced coupling with the right middle frontal gyrus and precentral gyrus. (c) Area 28/34_L (EC) demonstrated increased FC with multiple parietal and sensorimotor areas, including the right precentral gyrus, postcentral gyrus, paracentral lobule, superior parietal lobule (SPL), and precuneus. (d) Similarly, the left pulvinar nucleus exhibited enhanced connectivity with the temporal and opercular regions, including the right Rolandic operculum, superior temporal gyrus, Heschl’s gyrus, insula, and superior temporal pole. In contrast, the other seed ROI (intraparietal area 7_R) exhibited reduced FC with other brain regions, specifically shown as decreased coupling with the bilateral posterior cingulate cortex (PCC).

Network-level mapping based on Yeo parcellation demonstrated that these effects were distributed across 6 of the 7 canonical brain networks, with the DMN showing the most extensive involvement, whereas the ventral attention network was not implicated (Table 2 and Fig. 5F and G).

### Analysis of TMS–EEG changes

fMRI reveals enhanced static spatial connectivity between the DMN and high-order cognitive networks. However, higher-order cognition often relies on millisecond-level dynamic information integration. To further investigate whether tNIRS improves the temporal dynamic transmission efficiency of brain networks, we employed TMS–EEG to study the strength and efficiency of instantaneous information transmission in the brain.

**Fig. 6.**
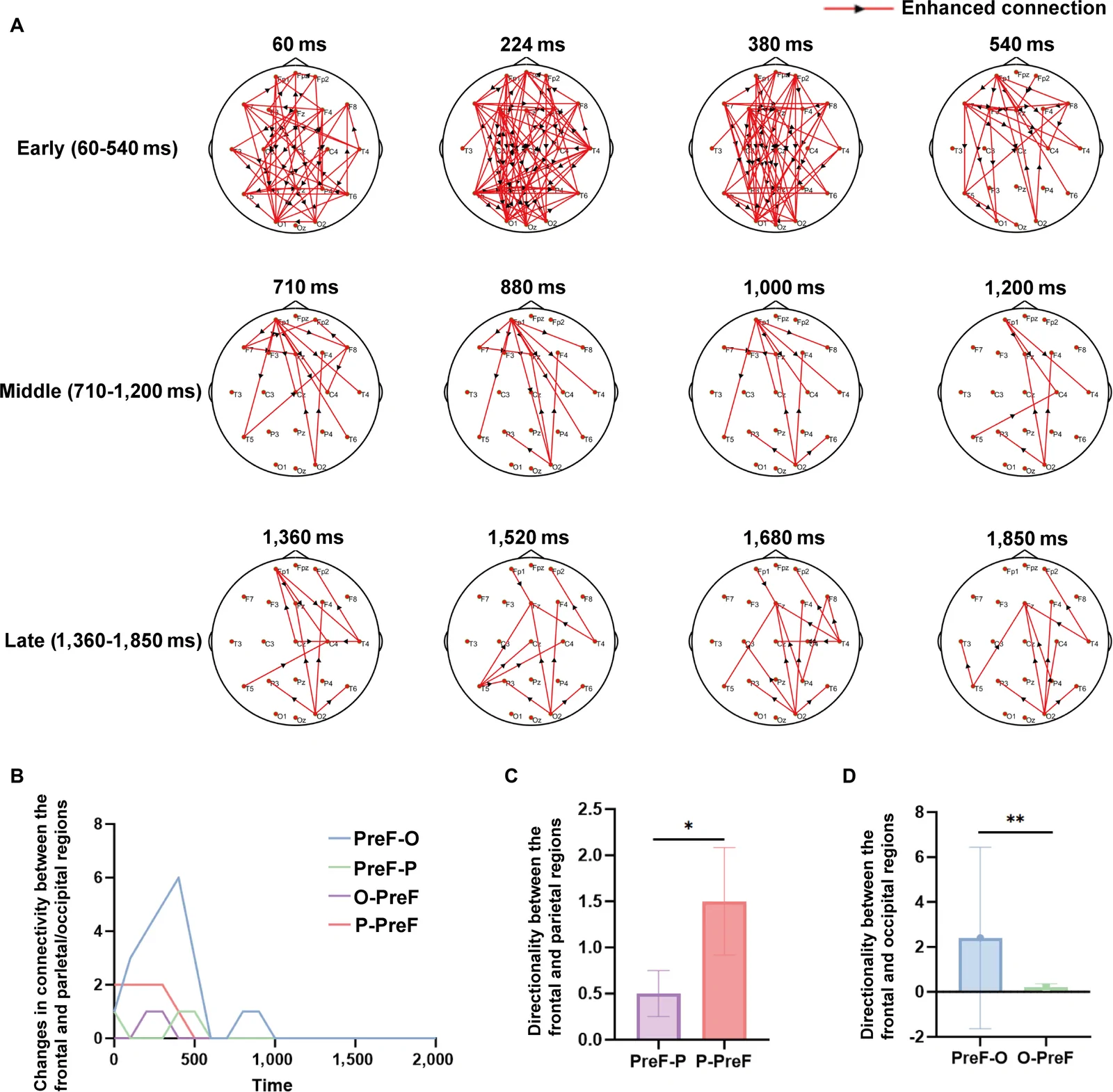
TMS–EEG connectivity changes in Alzheimer’s disease patients before and after tNIRS. (A) Connectivity changes after pre–post *t* tests. Time (milliseconds): After single-pulse TMS. Early, 60 to 540 ms; middle, 710 to 1,200 ms; late, 1,360 to 1,850 ms. Red line, enhanced connection; black arrows, direction of information flow. (B) Frontal and parieto-occipital connectivity changes over time after *t* tests. (C) Statistical analysis of connection directionality between prefrontal and parietal regions. (D) Statistical analysis of connection directionality between prefrontal and occipital regions. PreF, prefrontal cortex; P, parietal cortex; O, occipital cortex. *The *P* value was obtained using 2-sample *t* test, *P* < 0.05.

We discovered that, compared with baseline, patients with AD exhibited enhanced extensive neural connections in various brain regions after stimulation. In the early phase (60 to 540 ms), global enhancement peaked. In the mid-phase (710 to 1,200 ms), enhancement was concentrated in the left prefrontal cortex (the dominant outflow hub), right occipital lobe, and both temporal regions, and significant outflow from the occipital areas was also observed. In the late phase (1,360 to 1,850 ms), the overall connectivity strength resembled that of the mid-phase, but the occipital outflow had increased while the prefrontal outflow had declined. No connectivity reduction was detected after stimulation, indicating globally enhanced brain activity following tNIRS (Fig. 6A). Combining these results with the fMRI FC results, we analyzed meaningful TMS–EEG connections and directions in the frontal, parietal, and occipital regions. We discovered significant early-phase information flow from the parietal to the prefrontal regions and from the prefrontal to the occipital regions but no significant directional coupling between the parietal and occipital regions (Fig. 6B to D).

**Fig. 7.**
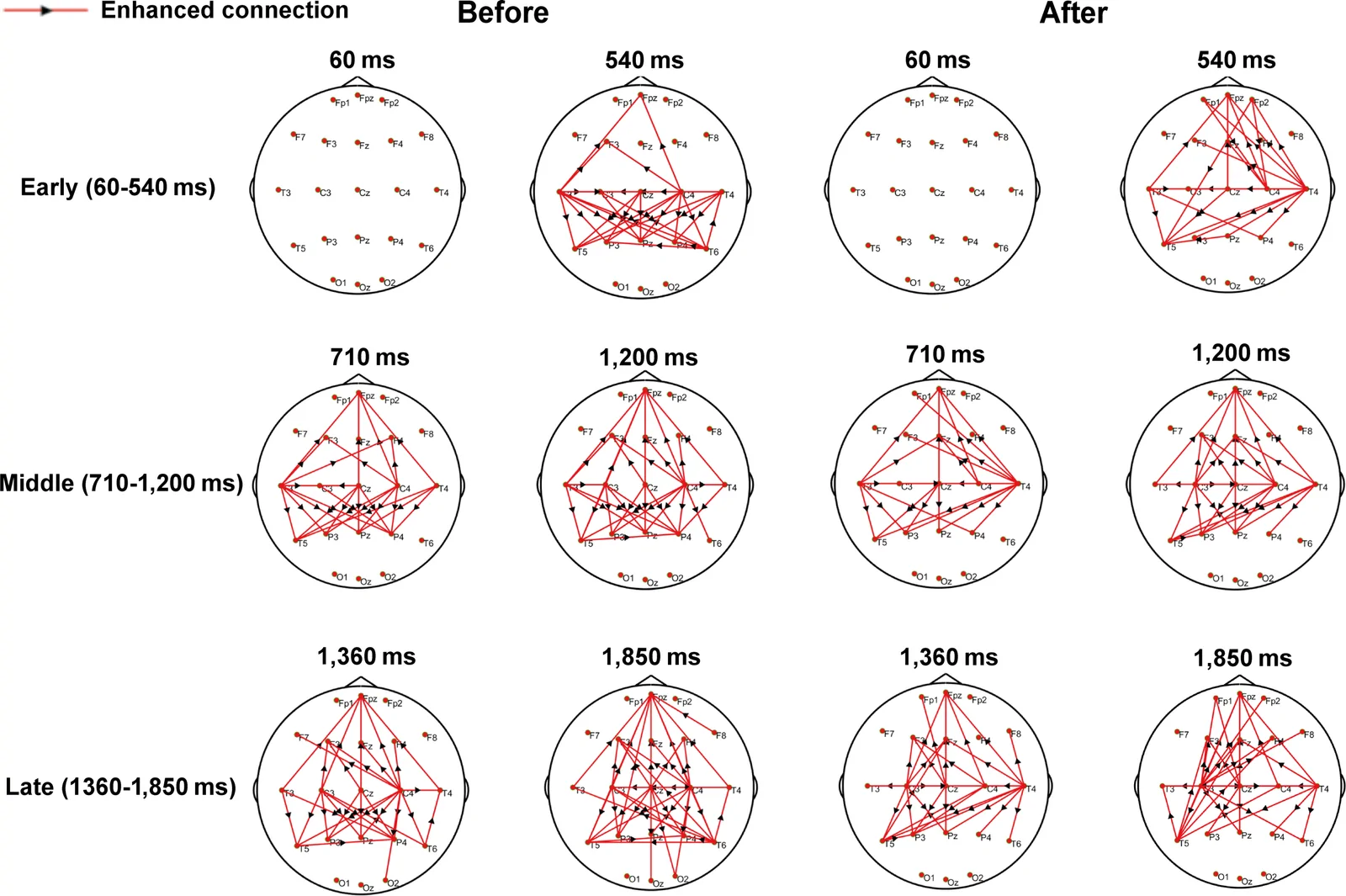
TMS–EEG network patterns over time in Alzheimer’s disease patients before and after tNIRS. Time (milliseconds): After single-pulse TMS. Early, 60 to 540 ms; middle, 710 to 1,200 ms; late, 1,360 to 1,850 ms. Red line, enhanced connection; black arrows, the direction of information flow.

Consistent with this, we further analyzed the reorganization pattern of the top 10% of FC strength before and after stimulation. Following stimulation, the brain’s FC underwent significant reorganization, with notably enhanced connections between the frontal lobe (electrodes Fz, Fp1, Fp2, F3) and temporal lobe (T4, T5, T3), as well as between the central region (C3, C4) and the temporal lobe (T4, T5) (Fig. 7).

## Discussion

The results of this study confirm that tNIRS that targets the AG ameliorates cognitive deficits and neuropsychiatric symptoms in patients with AD. Compared with the pretreatment baseline, patients with AD exhibited post-tNIRS improvements in global cognition, memory, language, attention, executive functions, and neuropsychiatric behaviors. The sham group exhibited negligible clinical efficacy. These findings indicate a significant short-term therapeutic efficacy of the tNIRS intervention. Post-tNIRS FC obviously increased within the DMN and among higher-order cognitive networks (DMN, DAN, FPN, and LN). Increased connectivity was observed between the temporal region within the DMN and the pulvinar region in the DAN, whereas connectivity was reduced between the posterior cingulate region in the DMN and the intraparietal area in the DAN. TMS–EEG revealed enhanced long-range (anterior–posterior and interhemispheric) connections in time-varying networks, along with improved information transmission efficiency in key hubs. These findings offer preliminary insights into the tNIRS-related mechanisms that may contribute to cognitive improvement in patients with AD. This may accelerate the use of tNIRS in neuroscience research and in the clinical management of aging-related neurological disorders. Notably, improvements in MMSE, MoCA, DST, and BNT scores attenuated at the 60-d follow-up. This pattern is consistent with previous PBM studies reporting cognitive declines after treatment cessation, suggesting that a single 15-d tNIRS course may be time-limited. Longer protocols (e.g., 4 months) have been associated with more sustained effects. Future studies should explore maintenance schedules and extended follow-up to optimize long-term benefits.

Our neuropsychological assessments, using multidimensional scales, demonstrated that bilateral AG-targeted tNIRS not only improved memory function in patients with AD but also enhanced performance across additional cognitive domains, including attention, executive function, and language processing. Critically, this intervention elevated global cognitive capacity. After the 15-d intervention, the intervention group exhibited significant cognitive improvements compared with baseline in the MMSE, MoCA, and ADAS-cog scores, reflecting overall cognitive-related correlations. Of note, these 3 scales consist of sections that test visuospatial ability, cognitive ability, and attention, such as clock-drawing tests, cube-drawing tests, and copying of simple graphical objects. The tNIRS group exhibited significant enhancements in these abilities by day 15, which may be related to the stimulation of both AG. A large amount of evidence supports the notion that the parietal cortex participates in spatial orientation and navigation. Using specialized tests of memory and attention (AVLT and DST), visuospatial and executive function (TMT), and language ability (BNT), we observed that participants in the tNIRS group had more significant improvements than those in the sham stimulation group.

Furthermore, tNIRS reportedly has the potential to ameliorate neuropsychiatric symptoms in patients with dementia [51]. Our research confirmed that potential, as indicated by the NPI results. Some of our participants exhibited abnormal mental behavior in the baseline NPI test, which is a burden to caregivers; however, by day 15, they exhibited improvements. Moreover, no adverse events occurred among the participants during the experimental procedure. These results suggest that tNIRS induces clinically significant changes, highlighting its potential utility to improve cognitive ability, executive function, and neuropsychiatric behavior in early-stage AD.

The AG was previously identified as a central parietal hub of the DMN associated with AD, exhibiting an integrative role in cross-regional communication [52,53]. From an optical perspective, selecting the 810-nm wavelength for targeted irradiation of the bilateral AG holds significant importance for quantitatively investigating the spatial heterogeneity of biological tissues. Recent optical biomedicine studies similarly show that AI-powered nonlinear optical imaging can quantify diabetic bone tissue heterogeneity, whereas frequency-specific tPBM may activate complementary glial mechanisms for neurovascular protection and amyloid beta clearance in AD [54,55]. The fundamental prerequisite for tNIRS therapy to exert clinical neuromodulatory effects is that a sufficient dose of photons must penetrate the scalp and cranial barriers to reach deep regions of the brain parenchyma. In this study, we integrated clinical observations with optical simulations using the MCVM to establish a physical foundation for the efficacy of 810-nm tNIRS. Based on a high-precision anatomical human head model, the MCVM simulations confirmed that 810-nm near-infrared light successfully achieved effective deep photon transmission due to its distinct physical properties of low absorption and high scattering within brain tissue. Furthermore, the simulations demonstrated that upon penetrating the skull, the photons generated a widespread optical scattering field within the targeted bilateral AG. Ultimately, these MCVM optical simulation results validate the scientific rationale for utilizing the 810-nm wavelength to precisely target and irradiate the bilateral AG.

Regarding the mechanisms underlying cognitive impairment in AD, the fMRI analysis comparing HC and the AD group revealed that, consistent with existing literature, brain regions with altered ALFF involved the DMN, FPN, and DAN, while ReHo alterations were primarily concentrated in core hubs of the DMN. These findings support the association between higher-order cognitive network dysfunction and AD pathogenesis, suggesting that such networks may serve as key targets for interventions like tNIRS neuromodulation aimed at restoring network function. Following 810-nm tNIRS that targeted both AG, our FC analyses revealed obviously enhanced intra-network connectivity within the DMN. Additionally, we detected region-specific connectivity enhancement across 9 network pairs: DMN–FPN/DAN/LN/visual network (VN)/sensorimotor network (SMN), LN–FPN/SMN, and DAN–FPN/SMN. In addition, increased connectivity was observed between the temporal region within the DMN and the pulvinar region in the DAN, whereas connectivity was reduced between the posterior cingulate region in the DMN and the intraparietal area in the DAN. DMN, DAN, FPN, and LN have been validated as regulators of higher-order cognition. The outcomes observed in this study, primarily characterized by enhanced intra-DMN connectivity and alterations in connectivity across multiple networks, appear to be closely associated with the integrative capacity of AG [56,57]. Thus, tNIRS may achieve these effects through bilateral AG stimulation alone, reaffirming the AG as a pivotal target for future tNIRS-based AD therapies.

Cognitive networks undergo preferential degradation in AD. This manifests as aberrant connectivity within and between these networks [58]. Degradation of intra-DMN connectivity is closely associated with AD progression [59,60]. We observed enhanced connectivity between orbital area 12/47_R and both precunei within the medial core subsystem of the DMN. The medial core subsystem may be involved in the consolidation and retrieval of episodic memory [61]. Aberrations in FC among higher-order cognitive networks (DMN, LN, and FPN) in patients with AD primarily manifest as segregation [39–42]. The EC within the LN serves as a relay hub in the bidirectional hippocampo-neocortical circuit, mediating memory integration. Our results demonstrated enhanced connectivity between the EC and precuneus in the DMN, which correlates with improved episodic memory [62]. As part of the neocortex, the SPL integrates spatiotemporal information and participates in memory retrieval [63–67]. We observed enhanced connectivity between the EC and SPL in the FPN, facilitating memory efficiency. Furthermore, we identified a strengthened connectivity between the parahippocampal gyrus in the medial temporal subsystem of the DMN and the middle frontal gyrus in the FPN [68]. Our preliminary findings suggest that tNIRS targeting the AG may contribute to enhanced connectivity within the DMN and among higher-order cognitive networks, which may result in improvements in cognitive function.

Notably, we observed weakened FC between the SPL in the DAN and the PCC in the DMN. As part of the medial core subsystem, the PCC mediates stimulus-independent thought and self-referential processing. In contrast, the SPL engages in top-down spatial attention control and exhibits antagonistic interactions with the DMN [69]. The attenuated SPL–PCC connectivity aligns with their antagonistic relationship and correlates with improved performance during attentionally demanding tasks [69].

Conversely, enhanced connectivity was detected between the left pulvinar region of the thalamus in the DAN and the right lateral temporal cortex/temporal pole in the DMN. The temporal regions belong to the dorsomedial subsystem and are implicated in social cognition. The thalamus is a subcortical hub that plays a critical role in attentional selection [70]. Their strengthened coordination facilitates an endogenous attentional focus toward behavioral goals [71]. We hypothesize that these DMN–DAN dynamics enhances the selective prioritization of internal/external cognition, thereby improving execution efficiency, accuracy, and behavioral patience. This may explain why tNIRS-treated participants exhibited increased task compliance during day 15 assessments and superior NPI score improvements compared with sham controls. This study suggests that tNIRS targeting of the AG may improve cognitive function in AD by modulating DMN–DAN connectivity-reducing attention-related SPL–PCC antagonism while enhancing pulvinar-temporal coordination, thereby improving executive efficiency and behavioral persistence.

The FPN acts as the brain’s central control switch, dynamically coordinating 2 key networks—the DMN, which handles internal thought, and the DAN, which manages external attention [72–75]. Our results demonstrated post-tNIRS enhancement of FPN-DMN and FPN-DAN connectivity, alongside optimized DMN–DAN functional coupling. These results suggest that tNIRS strengthens the regulatory role of the FPN, thereby facilitating cognitive control-related dynamic network interactions [76–78].

Complex cognition typically requires long-range integration across distributed brain networks [79,80]. TMS–EEG analyses revealed obviously enhanced global information flow after stimulation compared with baseline, with increased nodal activity predominantly observed in the left prefrontal cortex, right occipital lobe, and both temporal regions during the middle-to-late phases, while central and parietal nodal engagement markedly decreased. The observed changes in the TMS–EEG network indicated enhanced processing efficiency for the same type of information within the same brain regions after tNIRS therapy. Post-intervention *t*-statistic maps demonstrated robust enhancement of long-range information flow between the hemispheres and anteroposterior brain regions. These findings suggest that an augmented cross-network integration capacity improves cognitive function in patients with AD, underscoring the pivotal role of long-range connections in cognitive processing.

The fMRI and TMS–EEG findings in our study are best interpreted as complementary evidence for network-level reconfiguration. fMRI provides high spatial-resolution information about connectivity changes across large-scale networks, while TMS–EEG provides high temporal-resolution information about directional information flow along the same fronto-parieto-occipital axis. Although formal cross-modal correlation analysis was not performed, the convergence of evidence from both modalities—despite capturing different aspects of neural activity—supports the interpretation that tNIRS reshapes large-scale brain network dynamics.

Furthermore, the integration of local interregional connectivity may play a critical role in the facilitation of information processing [81,82]. FC analysis identified strengthened connectivity between the right orbitofrontal cortex and both cunei/the right superior occipital gyrus. This pattern engages the anterior, central, and posterior regions and involves both the anterior/posterior DMN subsystems and visual networks. Therefore, we aimed to analyze the specific mechanism of tNIRS-enhanced brain information processing by quantifying the directional interactions among these 3 brain regions. Directional statistics applied to TMS–EEG pattern maps demonstrated significant early-phase information flow from the parietal to the prefrontal regions and from the prefrontal to the occipital regions, but no significant directional coupling between the parietal and occipital regions. This indicates that tNIRS altered directional network dynamics, strengthening the parietal–prefrontal–occipital neural circuitry in patients with AD. Such directional enhancement reflects (a) strengthened feedback from the posterior DMN hub (parietal cortex) to the anterior DMN (prefrontal cortex) and (b) improved top-down modulation from higher-order prefrontal regions to the lower-order visual cortex, thereby optimizing information integration, attentional control, and cognitive improvement [83]. Similarly, analysis of the key nodes (top 10%) in information flow strength revealed that after intervention, the pivotal hubs for information outflow and inflow were more concentrated in the prefrontal and temporal regions. This indicates that tNIRS treatment enhanced the effective participation of local nodes within the prefrontal and temporal networks during cognitive processing. These findings confirm that 810-nm tNIRS that targets the AG increases the number of functional network hubs and enhances internodal integration efficiency, ultimately improving information processing capacity.

This study breaks away from the traditional multi-target intervention paradigm of tNIRS for the treatment of AD. By using a randomized, controlled, blinded design, we confirmed that the AG serve as pivotal targets for 810-nm tNIRS. This single-target stimulation elicits coordinated responses across multiple brain regions. Combined analyses of resting-state FC and dynamic TMS–EEG demonstrated that this paradigm optimized intra-DMN and internetwork connectivity among higher-order cognitive networks. It obviously enhances dynamic information transmission across large-scale networks, thereby improving the operational efficiency of the brain for information reception, processing, and feedback. These findings suggest that the mechanisms effectively ameliorate multi-domain cognitive dysfunction in patients with AD.

This study also had certain limitations. First, the relatively small sample size limits the generalizability of our findings and precludes detailed subgroup or mediation analyses. Future large-scale, multi-center studies are warranted to confirm these preliminary observations. Second, no hierarchical analysis was conducted, no task state was assessed, and this study was focused only on the brain network mechanism. Third, the angular gyri were localized using the EEG 10-20 system (P3/P4) rather than neuronavigation, which may introduce targeting variability. Future studies should adopt MRI-guided neuronavigation to improve precision. Fourth, fMRI and TMS–EEG data were collected only from the active group, precluding exclusion of time, practice, or placebo effects on network changes. Thus, our mechanistic interpretations remain preliminary and hypothesis-generating, not causal. Future sham-controlled studies should include longitudinal neuroimaging in both groups to enable robust causal inferences. Fifth, due to the limited fMRI subsample size, we did not perform a formal mediation analysis to establish a causal link between network changes and cognitive improvements—such analyses typically require much larger samples for reliable parameter estimation. Future studies with larger samples should perform formal mediation analyses to directly test whether specific network changes mediate the cognitive benefits of tNIRS.

## Materials and Methods

### Participant recruitment

The research protocol was approved by the Research Ethics Committee of the First Hospital of Hebei Medical University [(2024) Research Review No. (110)] and is in line with the Declaration of Helsinki. All patients or family members agree to participate in this study and sign informed consent. This prospective, randomized, sham-controlled study included 36 right-handed patients (12 males and 24 females) with clinically diagnosed AD who were selected from the First Hospital of Hebei Medical University as the experimental group, and 16 healthy older adults were selected in the community as normal control groups.

Based on previous findings, sample size estimation was performed using G*Power 3.1 software. Assuming a medium effect size (Cohen’s *f* = 0.25), a statistical power of 80%, and a significance level of 5%, a minimum of 14 participants per group was required. Accounting for a potential dropout rate of 20%, we planned to recruit at least 34 participants in total. Ultimately, 36 patients were enrolled, with 18 in each group.

Patients who met the following requirements were included in the experimental group: (a) provided informed consent; (b) literate Han Chinese, 50 to 80 years old, right-handed; (c) have at least 6 years of education; (d) diagnosis of mild AD according to the core diagnostic criteria of the National Institute on Aging and the Alzheimer’s Association (NIA-AA) guidelines [84]; (e) CSF Aβ levels decreased and total tau or P-tau levels increased or amyloid positron emission tomography showed increased tracer retention [85]; (f) the overall score of the CDR was 0.5 or 1 point [86], the MMSE ≥ 18 points [87], and the MoCA < 26 points [88]; (g) those who had not taken drugs for the treatment of AD (donepezil, memantine hydrochloride, rivastigmine tartrate, or others) and did not take other drugs that affect cognitive function during and within 8 d after giving near-infrared light stimulation, or had been taking drugs regularly for more than 1 year and did not need to adjust the type and dosage of drugs during the light stimulation.

Exclusion criteria for all participants included (a) patients with other neurological and psychiatric diseases and other serious diseases such as heart, liver, lung, and kidney diseases; (b) those who are equipped with metal inserts such as pacemakers, coronary stents, and aneurysm clips; and (c) those who take benzodiazepines or have a history of drug abuse.

Patients who meet the following requirements were included in the HC group: (a) right-handed seniors between the ages of 55 and 80 who can speak Mandarin; (b) no mild cognitive impairment, dementia, or other major psychiatric or neurological diseases; and (c) the absence of other disorders that may cause cognitive decline.

Baseline demographic and clinical characteristics were collected for all participants before the start of the experiment. To ensure that subjects were unaware of each other’s group assignment and to prevent communication between them, the relevant parties, including subjects, evaluators, and statisticians, were blinded to group allocation until the statistical analysis report was generated. The near-infrared light stimulation was administered by independent personnel.

### MCVM

MCVM is an advanced optical algorithmic tool that simulates the propagation behavior of photons within complex biological tissues using a probabilistic approach. Unlike traditional Monte Carlo models that are largely restricted to multi-layered tissue structures, MCVM is highly adaptable to arbitrary 3D voxelized media, making it particularly suitable for complex, high-resolution human anatomical models. During the simulation, a massive number of photons are injected into the target tissue model. The model tracks the stochastic transport of each individual photon as it undergoes random scattering or absorption events by particles in the medium, continuing until the photons are either completely absorbed by the tissue or transmitted out of the tissue surface. Crucially, MCVM processes these interactions strictly at the voxel level; each computational substep is tied to a single voxel and its specific optical properties, namely, the absorption coefficient and scattering coefficient. This precise localization ensures accurate quantification of photon energy attenuation.

### Neuropsychological assessment

Cognitive function is assessed using ADAS-cog [89], MMSE [87], and MoCA [88]. Memory is evaluated with the AVLT [90]. Attention is assessed via the DST [91]. Language ability is measured using the BNT [92]. Visuospatial and executive functions are evaluated with the TMT [93]. Behavioral and psychological changes are assessed with the NPI [94]. Safety is monitored by tracking the incidence and severity of adverse events, which are immediately reported to and managed by experienced physicians.

All assessments are conducted by trained research assistants. The primary outcomes are MMSE, MoCA, and ADAS-cog scores. Secondary outcomes include results from the NPI, AVLT, DST, TMT, and other tests, along with adverse events. Assessments are performed at baseline, 15 d post-stimulation (D15), and 60 d post-intervention (follow-up, D75).

### Resting-state fMRI data acquisition and preprocessing

Participants with no contraindications to MRI underwent scans in the First Hospital of Hebei Medical University. Structural MRI and resting-state functional MRI were obtained on a 3-T MAGNETOM Trio scanner (Siemens, Erlangen, Germany). T1 structural images were acquired using a 3D magnetized preparation fast gradient echo (3DMPRAGE) sequence with the following parameters: repetition time (TR) = 1,690 ms, echo time (TE) = 2.56 ms, flip angle (FA) = 12°, field of view (FOV) = 256 × 256 mm^2^, matrix (MATRIX) = 256 × 256, voxel size = 1 mm isotropic, 176 gapless staggered sagittal slices. For resting-state fMRI with a duration of 6 min, multi-band echo plane imaging (EPI) with the following parameters was used: TR = 2,000 ms, TE = 30 ms, FA = 83°, FOV = 224 × 224 mm^2^, MATRIX = 64 × 64, number of scanned layers = 36 pieces; slice thickness = 3 mm; scan spacing = 1 mm.

The preprocessing of the data was carried out in MATLAB using the SPM, DPABI, and RESTplus software. First, the dcm2niigui functional tool was used in MRIcron to convert the data acquired from clinical MRI in DICOM format into a NIFTI format, which can describe the pixel values and spatial coordinates of the pixels in detail and more accurately reflect the original metadata. The specific process of pretreatment is as follows: (a) The first 10 time points were removed to reduce environmental interference, participant-related factors, and the effects of initial magnetic field instability on imaging. (b) Origin correction was performed, and image orientation was adjusted to ensure that all images are compared and analyzed in the same anatomical orientation. (c) A spatial normalization process is performed to align the images with the criteria of the Montreal Neurological Institute, in which an image of the individual T1 structure is used as a reference. The T1 structure image is registered as its corresponding average functional image, which facilitates subsequent comparison of brain image data between different individuals. (d) The method of improving noise characteristics and signal accuracy is image smoothing with Gaussian*kernel (full width at half maximum = 6 mm). (e) Linear trend removal steps have been implemented to eliminate confounders. (f) Interference was mitigated, including removal of head motion effects, and signal regression. (g) Bandpass filter (0.01 to 0.08Hz) was applied to eliminate high-frequency noise and low-frequency signal drift.

### TMS–EEG data acquisition

The patient was equipped with an electrode cap with 32 TMS electrodes (Greentech Medical, Wuhan, China), and the data were digitized with a magnetic field-enabled EEG amplifier at a sampling rate of 1,024 Hz (Yunshen Technology Co. Ltd., Beijing, China). TMS–EEG data were collected from 18 patients under active stimulation before and after tNIRS intervention. All AD patients underwent resting motor threshold (RMT) assessment, which was defined as the minimum stimulation intensity required to elicit motor evoked potentials with a peak-to-peak amplitude of at least 50 μV in the resting abductor pollicis brevis muscle in 10 consecutive trials of the abductor pollicis brevis at rest [95]. The right P4 was stimulated with 140 single-pulse TMS (sTMS) (MagPro R30, Tonica Elektronik A/S, Farum, Denmark) and 90% RMT (P3/P4 on the participant’s scalp, based on the international 10-20 system), with each stimulus delivered at 4-s intervals.

### Statistical analysis of population characteristics

Statistical analysis was performed using SPSS 22.0 (SPSS Inc., IL, USA) and GraphPad Prism 10. The continuous variables between the 2 groups were analyzed by independent samples *t* test, the continuous variables between multiple groups were analyzed by Welch’s analysis of variance (ANOVA), and the chi-square test was performed for categorical variables. The Shapiro–Wilk test was used to evaluate the normality of the main and secondary indicators in the neuropsychological assessment. The Friedman test and Wilcoxon were used to evaluate the differences within the group, and after Bonferroni correction (α = 0.05/3 ≈ 0.0167), *P* < 0.0167 was considered statistically significant. The generalized estimation equation was used to assess the differences between groups, and the time × group interaction *P* < 0.05 was considered statistically significant. Effect sizes were reported as Kendall’s *W* (Friedman tests), *r* = *Z*/√*n* (within-group Wilcoxon tests), and *r* = *Z*/√*N* (between-group Mann–Whitney *U* tests), with detailed results provided in Tables S1 to S5.

### fMRI analysis

Due to head movement during fMRI data acquisition in 2 subjects, data from only 16 AD patients were available for subsequent analysis.

The ALFF was extracted by RESTplus software in MATLAB 2019a from the pretreated normal control group and the tNIRS group before treatment, and the time series of each voxel was converted into the frequency domain to obtain the power spectrum by using the fast Fourier transform, and the square root of the power spectrum was calculated and averaged in the range of 0.01 to 0.08 Hz.

ReHo reflects the synchronization of blood oxygen level-dependent (BOLD) signal time series between adjacent voxels, representing the capability of local functional integration. RESTplus calculates the temporal consistency of each voxel with its 26 neighboring voxels using Kendall’s coefficient of concordance to generate the raw ReHo map. Subsequently, the ReHo map undergoes Fisher-*Z* transformation to improve the normality of the data distribution. Finally, spatial smoothing is applied with a Gaussian kernel of 6-mm FWHM, resulting in a smoothed and standardized ReHo map (referred to as the SzKccReHo metric) for subsequent statistical analysis. This process effectively reduces partial volume effects and enhances the signal-to-noise ratio and normality of the data.

Subsequently, the statistical function in DPABI software was used to perform 2-sample *t* tests on the ALFF and ReHo results of the 2 groups, respectively, generating .nil files. Gaussian random field correction was then applied in the Viewer function of the software. Brain regions meeting the criteria of voxel-level threshold *P* < 0.001 and cluster-level threshold *P* < 0.05 for ALFF, as well as voxel-level threshold *P* < 0.005 and cluster-level threshold *P* < 0.05 for ReHo, were selected.

Based on the ALFF and ReHo findings of healthy individuals and pre-stimulated patients with AD, we identified seed points for FC. These seed points were combined with brain regions associated with cognitive function. Consequently, we established the presence of the prefrontal region, cingulate gyrus, cuneiform lobe, hippocampus, parahippocampal region, thalamus, and striatal region. For functional ligation analysis of resting-state functional nuclear magnetic resonance (NMR), we used 2 different methods: (a) map-based ROI-to-ROI functional ligation analysis and (b) seed-to-voxel analysis using a priori selections derived from ROI-to-ROI results. In the map-based ROI-to-ROI analysis, the preprocessed data were parcellated into 246 ROIs using the Brainnetome Atlas; the mean BOLD (blood oxygen level-dependent) time series was extracted from all voxels within each ROI, and pairwise Pearson correlation coefficients between ROI time series were calculated and Fisher z-transformed to generate an individual connectivity matrix [96]. Similarly, in seed-to-voxel analysis, a correlation map on the whole brain is generated by extracting the BOLD signal from the seed ROI, calculating the correlation coefficient between that signal and the signal from all other brain voxels, and *z*-transforming it. After ROI-to-ROI and voxel-based analyses at the subject level, the general linear model was fitted using all corresponding intra-subject pairwise *z*-conversion correlation coefficient measurements. Then, ROI-to-ROI analysis was performed with a voxel threshold *P* < 0.001 and a cluster-level threshold *P* < 0.05 (GRF corrected).

### TMS–EEG analysis

MATLAB (R2019b, MathWorks, USA) was selected for EEG signal analysis (3 to 30 Hz bandpass filter) and EEG data segmentation. First, each TMS event is labeled, and the time point corresponding to the label peak is set to time “0”. The corresponding data for each magnetic stimulus 500 ms before and after 2,000 ms were extracted. Then, a time-varying multivariate adaptive autoregressive (tv-MVAAR) model was developed and the adapted directed transfer function matrix was calculated. The rows and columns in these matrices represent the nodes, while the matrix elements at the intersection of rows i and column j represent information about the connections between the nodes. When plotting the brain network, a *t* test was performed on all connections at each time point before and after stimulation to investigate changes in global information transfer efficiency induced by the intervention. Meanwhile, the top 10% of enhanced connections in strength at each time point were selected to examine the reorganization patterns of local nodes before and after the intervention [97–100].

## Ethical Approval

This study was approved by the Research Ethics Committee of the First Hospital of Hebei Medical University [(2024) Research Review No. (110)] and was performed in accordance with the Declaration of Helsinki. All participants signed written informed consent documents before participating in the study.

## Data Availability

The datasets generated and analyzed during the current study are available from the corresponding authors upon reasonable request.

## References

[1] Nichols E, Steinmetz JD, Vollset SE, Fukutaki K, Chalek J, Abd-Allah F, Abdoli A, Abualhasan A, Abu-Gharbieh E, Akram TT, et al. Estimation of the global prevalence of dementia in 2019 and forecasted prevalence in 2050: An analysis for the global burden of disease study 2019. Lancet Public Health. 2022;7(2):e105–e125. 10.1016/S2468-2667(21)00249-8 PMC8810394 34998485

[2] Alzheimer’s Association. 2016 Alzheimer’s disease facts and figures. Alzheimers Dement. 2016;12(4):459–509. 10.1016/j.jalz.2016.03.001 27570871

[3] Zhang Y, Chen H, Li R, Sterling K, Song W. Amyloid β-based therapy for Alzheimer’s disease: Challenges, successes and future. Signal Transduct Target Ther. 2023;8(1):248. 10.1038/s41392-023-01484-7 PMC10310781 37386015

[4] Cummings J, Zhou Y, Lee G, Zhong K, Fonseca J, Cheng F. Alzheimer’s disease drug development pipeline: 2023. Alzheimers Dement. 2023;9(2): Article e12385. 10.1002/trc2.12385 PMC10210334 37251912

[5] Baik JS, Lee TY, Kim NG, Pak K, Ko S-H, Min JH, Shin Y-I. Effects of Photobiomodulation on changes in cognitive function and regional cerebral blood flow in patients with mild cognitive impairment: A pilot uncontrolled trial. J Alzheimers Dis. 2021;83(4):1513–1519. 10.3233/JAD-210386 34420956

[6] Huang N, Yao D, Jiang W, Wei C, Li M, Li W, Mu H, Gao M, Ma Z, Lyu J, et al. Safety and efficacy of 630-nm red light on cognitive function in older adults with mild to moderate Alzheimer’s disease: Protocol for a randomized controlled study. Front Aging Neurosci. 2020;12:143. 10.3389/fnagi.2020.00143 PMC7253693 32528273

[7] Pruitt T, Wang X, Wu A, Kallioniemi E, Husain MM, Liu H. Transcranial Photobiomodulation (tPBM) with 1,064-nm laser to improve cerebral metabolism of the human brain in vivo. Lasers Surg Med. 2020;52(9):807–813. 10.1002/lsm.23232 PMC7492377 32173886

[8] Salehpour F, Cassano P, Rouhi N, Hamblin MR, De Taboada L, Farajdokht F, Mahmoudi J. Penetration profiles of visible and near-infrared lasers and light-emitting diode light through the head tissues in animal and human species: A review of literature. Photobiomodul Photomed Laser Surg. 2019;37(10):581–595. 10.1089/photob.2019.4676 31553265

[9] Jagdeo JR, Adams LE, Brody NI, Siegel DM. Transcranial red and near infrared light transmission in a cadaveric model. PLOS ONE. 2012;7(10): Article e47460. 10.1371/journal.pone.0047460 PMC3471828 23077622

[10] Tedford CE, DeLapp S, Jacques S, Anders J. Quantitative analysis of transcranial and intraparenchymal light penetration in human cadaver brain tissue. Lasers Surg Med. 2015;47(4):312–322. 10.1002/lsm.22343 25772014

[11] Lim L. Traumatic brain injury recovery with Photobiomodulation: Cellular mechanisms, clinical evidence, and future potential. Cells. 2024;13(5):385. 10.3390/cells13050385 PMC10931349 38474349

[12] Morse PT, Goebel DJ, Wan J, Tuck S, Hakim L, Hüttemann CL, Malek MH, Lee I, Sanderson TH, Hüttemann M. Cytochrome c oxidase-modulatory near-infrared light penetration into the human brain: Implications for the noninvasive treatment of ischemia/reperfusion injury. IUBMB Life. 2021;73(3):554–567. 10.1002/iub.2405 PMC8819601 33166061

[13] Cury V, Moretti AI, Assis L, Bossini P, de Souza Crusca J, Neto CB, Fangel R, De Souza HP, Hamblin MR, Parizotto NA. Low level laser therapy increases angiogenesis in a model of ischemic skin flap in rats mediated by VEGF, HIF-1α and MMP-2. J Photochem Photobiol B Biol. 2013;125:164–170. 10.1016/j.jphotobiol.2013.06.004 PMC3759230 23831843

[14] Wang M, Yan C, Li X, Yang T, Wu S, Liu Q, Luo Q, Zhou F. Non-invasive modulation of meningeal lymphatics ameliorates ageing and Alzheimer’s disease-associated pathology and cognition in mice. Nat Commun. 2024;15(1):1453. 10.1038/s41467-024-45656-7 PMC10873306 38365740

[15] Zinchenko E, Navolokin N, Shirokov A, Khlebtsov B, Dubrovsky A, Saranceva E, Abdurashitov A, Khorovodov A, Terskov A, Mamedova A, et al. Pilot study of transcranial photobiomodulation of lymphatic clearance of beta-amyloid from the mouse brain: Breakthrough strategies for non-pharmacologic therapy of Alzheimer’s disease. Biomed Opt Express. 2019;10(8):4003–4017. 10.1364/BOE.10.004003 PMC6701516 31452991

[16] Lu Y, Wang R, Dong Y, Tucker D, Zhao N, Ahmed ME, Zhu L, Liu TC, Cohen RM, Zhang Q. Low-level laser therapy for beta amyloid toxicity in rat hippocampus. Neurobiol Aging. 2017;49:165–182. 10.1016/j.neurobiolaging.2016.10.003 PMC5458630 27815990

[17] Yang L, Wu C, Parker E, Li Y, Dong Y, Tucker L, Brann DW, Lin HW, Zhang Q. Non-invasive photobiomodulation treatment in an Alzheimer disease-like transgenic rat model. Theranostics. 2022;12(5):2205–2231. 10.7150/thno.70756 PMC8899582 35265207

[18] Karu TI. Multiple roles of cytochrome c oxidase in mammalian cells under action of red and IR-A radiation. IUBMB Life. 2010;62(8):607–610. 10.1002/iub.359 20681024

[19] Karu TI. Mitochondrial signaling in mammalian cells activated by red and near-IR radiation. Photochem Photobiol. 2008;84(5):1091–1099. 10.1111/j.1751-1097.2008.00394.x 18651871

[20] Karu TI, Pyatibrat LV, Kalendo GS. Photobiological modulation of cell attachment via cytochrome c oxidase. Photochem Photobiol Sci. 2004;3(2):211–216. 10.1039/b306126d 14872239

[21] Kashiwagi S, Morita A, Yokomizo S, Ogawa E, Komai E, Huang PL, Bragin DE, Atochin DN. Photobiomodulation and nitric oxide signaling. Nitric Oxide. 2023;130:58–68. 10.1016/j.niox.2022.11.005 PMC9808891 36462596

[22] Yang Q, Qu X, Sheng C, Zhao X, Chen G, Wang X, Li Y, Du W, Wang X, Sun Y, et al. Transcranial photobiomodulation improves functional brain networks and working memory in healthy older adults: An fNIRS study. NeuroImage. 2025;316: Article 121305. 10.1016/j.neuroimage.2025.121305 40482941

[23] de Oliveira BH, Lins EF, Kunde NF, Salgado AS, Martins LM, Bobinski F, Vieira WF, Cassano P, Quialheiro A, Martins DF. Transcranial photobiomodulation increases cognition and serum BDNF levels in adults over 50 years: A randomized, double-blind, placebo-controlled trial. J Photochem Photobiol B. 2024;260: Article 113041. 10.1016/j.jphotobiol.2024.113041 39423445

[24] Tian F, Hase SN, Gonzalez-Lima F, Liu H. Transcranial laser stimulation improves human cerebral oxygenation. Lasers Surg Med. 2016;48(4):343–349. 10.1002/lsm.22471 PMC5066697 26817446

[25] Salehpour F, Hamblin MR, DiDuro JO. Rapid reversal of cognitive decline, olfactory dysfunction, and quality of life using multi-modality photobiomodulation therapy: Case report. Photobiomodul Photomed Laser Surg. 2019;37(3):159–167. 10.1089/photob.2018.4569 31050946

[26] Cheung M-C, Lee T-L, Sze SL, Chan AS. Photobiomodulation improves frontal lobe cognitive functions and mental health of older adults with non-amnestic mild cognitive impairment: Case studies. Front Psychol. 2022;13: Article 1095111. 10.3389/fpsyg.2022.1095111 PMC9871821 36704674

[27] Chao LL. Effects of home Photobiomodulation treatments on cognitive and behavioral function, cerebral perfusion, and resting-state functional connectivity in patients with dementia: A pilot trial. Photobiomodul Photomed Laser Surg. 2019;37(3):133–141. 10.1089/photob.2018.4555 31050950

[28] Kheradmand A, Donboli S, Tanjani PT, Farhadinasab A, Tabeie F, Qutbi M, Kordmir T. Therapeutic effects of low-level laser therapy on cognitive symptoms of patients with dementia: A double-blinded randomized clinical trial. Photobiomodul Photomed Laser Surg. 2022;40(9):632–638. 10.1089/photob.2021.0135 36126290

[29] Weiler M, Teixeira CV, Nogueira MH, de Campos BM, Damasceno BP, Cendes F, Balthazar ML. Differences and the relationship in default mode network intrinsic activity and functional connectivity in mild Alzheimer’s disease and amnestic mild cognitive impairment. Brain Connect. 2014;4(8):567–574. 10.1089/brain.2014.0234 PMC4202997 25026537

[30] Pan P, Zhu L, Yu T, Shi H, Zhang B, Qin R, Zhu X, Qian L, Zhao H, Zhou H, et al. Aberrant spontaneous low-frequency brain activity in amnestic mild cognitive impairment: A meta-analysis of resting-state fMRI studies. Ageing Res Rev. 2017;35:12–21. 10.1016/j.arr.2016.12.001 28017880

[31] Tosun D, Landau S, Aisen PS, Petersen RC, Mintun M, Jagust W, Weiner MW, Alzheimer’s Disease Neuroimaging Initiative. Association between tau deposition and antecedent amyloid-β accumulation rates in normal and early symptomatic individuals. Brain. 2017;140(5):1499–1512. 10.1093/brain/awx046 28334939

[32] Kalpouzos G, Eriksson J, Sjölie D, Molin J, Nyberg L. Neurocognitive systems related to real-world prospective memory. PLOS ONE. 2010;5(10): Article e13304. 10.1371/journal.pone.0013304 PMC2951914 20949046

[33] Seghier ML. The angular gyrus: Multiple functions and multiple subdivisions. Neuroscientist. 2013;19(1):43–61. 10.1177/1073858412440596 PMC4107834 22547530

[34] Petit L, Ali KM, Rheault F, Boré A, Cremona S, Corsini F, De Benedictis A, Descoteaux M, Sarubbo S. The structural connectivity of the human angular gyrus as revealed by microdissection and diffusion tractography. Brain Struct Funct. 2023;228(1):103–120. 10.1007/s00429-022-02551-5 35995880

[35] Chen H-F, Sheng X-N, Yang Z-Y, Shao P-F, Xu H-H, Qin R-M, Zhao H, Bai F. Multi-networks connectivity at baseline predicts the clinical efficacy of left angular gyrus-navigated rTMS in the spectrum of Alzheimer’s disease: A sham-controlled study. CNS Neurosci Ther. 2023;29(8):2267–2280. 10.1111/cns.14177 PMC10352882 36942495

[36] Hu Y, Jia Y, Sun Y, Ding Y, Huang Z, Liu C, Wang Y. Efficacy and safety of simultaneous rTMS-tDCS over bilateral angular gyrus on neuropsychiatric symptoms in patients with moderate Alzheimer’s disease: A prospective, randomized, sham-controlled pilot study. Brain Stimul. 2022;15(6):1530–1537. 10.1016/j.brs.2022.11.009 36460293

[37] Jones DT, Knopman DS, Gunter JL, Graff-Radford J, Vemuri P, Boeve BF, Petersen RC, Weiner MW, Jack CR Jr. Cascading network failure across the Alzheimer’s disease spectrum. Brain. 2016;139(Pt 2):547–562. 10.1093/brain/awv338 PMC4805086 26586695

[38] Anticevic A, Cole MW, Murray JD, Corlett PR, Wang X-J, Krystal JH. The role of default network deactivation in cognition and disease. Trends Cogn Sci. 2012;16(12):584–592. 10.1016/j.tics.2012.10.008 PMC3501603 23142417

[39] Zhao Q, Sang X, Metmer H, Lu J, Alzheimer’s Disease NeuroImaging Initiative. Functional segregation of executive control network and frontoparietal network in Alzheimer’s disease. Cortex. 2019;120:36–48. 10.1016/j.cortex.2019.04.026 31228791

[40] Wang K, Liang M, Wang L, Tian L, Zhang X, Li K, Jiang T. Altered functional connectivity in early Alzheimer’s disease: A resting-state fMRI study. Hum Brain Mapp. 2007;28(10):967–978. 10.1002/hbm.20324 PMC6871392 17133390

[41] Sun Y-W, Lyu X-Y, Lei X-Y, Huang M-M, Wang Z-M, Gao B. Association study of brain structure-function coupling and glymphatic system function in patients with mild cognitive impairment due to Alzheimer’s disease. Front Neurosci. 2024;18: Article 1417986. 10.3389/fnins.2024.1417986 PMC11320604 39139498

[42] Allen G, Barnard H, McColl R, Hester AL, Fields JA, Weiner MF, Ringe WK, Lipton AM, Brooker M, McDonald E, et al. Reduced hippocampal functional connectivity in Alzheimer disease. Arch Neurol. 2007;64(10):1482–1487. 10.1001/archneur.64.10.1482 17923631

[43] Zhang HQ, Chau ACM, Shea YF, Chiu PK, Bao YW, Cao P, Mak HK. Disrupted structural white matter network in Alzheimer’s disease continuum, vascular dementia, and mixed dementia: A diffusion tensor imaging study. J Alzheimers Dis. 2023;94(4):1487–1502. 10.3233/JAD-230341 37424470

[44] Baik K, Yang J-J, Jung JH, Lee YH, Chung SJ, Yoo HS, Sohn YH, Lee PH, Lee J-M, Ye BS. Structural connectivity networks in Alzheimer’s disease and Lewy body disease. Brain Behav. 2021;11(5): Article e02112. 10.1002/brb3.2112 PMC8119831 33792194

[45] Fox MD, Raichle ME. Spontaneous fluctuations in brain activity observed with functional magnetic resonance imaging. Nat Rev Neurosci. 2007;8(9):700–711. 10.1038/nrn2201 17704812

[46] Hernandez-Pavon JC, Veniero D, Bergmann TO, Belardinelli P, Bortoletto M, Casarotto S, Casula EP, Farzan F, Fecchio M, Julkunen P, et al. TMS combined with EEG: Recommendations and open issues for data collection and analysis. Brain Stimul. 2023;16(2):567–593. 10.1016/j.brs.2023.02.009 36828303

[47] Zhang B, Yang S, Piao M, Chang P, Li T. Effect of incidence sites on light distribution at different wavelengths during transcranial Photobiomodulation. Acad Radiol. 2026;33(5):1767–1780. 10.1016/j.acra.2025.04.076 40410107

[48] Yang S, Zhang B, Li M, Liu Z, Yang X, Li Y, Li T. Bridging animal and human: A 3D computational framework of light propagation in head models for transcranial photobiomodulation in Alzheimer’s disease. Brain-X. 2026;4(1): Article e70048.

[49] Guo J, Meng S, Su H, Zhang B, Li T. Non-invasive optical monitoring of human lungs: Monte Carlo modeling of photon migration in visible Chinese human and an experimental test on a human. Biomed Opt Express. 2022;13(12):6389–6403. 10.1364/BOE.472530 PMC9774858 36589576

[50] Li T, Li Y, Sun Y, Duan M, Peng L. Effect of head model on Monte Carlo modeling of spatial sensitivity distribution for functional near-infrared spectroscopy. J Innov Opt Health Sci. 2014;08(05):1550024.

[51] Zomorrodi R, Loheswaran G, Pushparaj A, Lim L. Pulsed near infrared transcranial and intranasal Photobiomodulation significantly modulates neural oscillations: A pilot exploratory study. Sci Rep. 2019;9(1):6309. 10.1038/s41598-019-42693-x PMC6474892 31004126

[52] Liang X, Zou Q, He Y, Yang Y. Coupling of functional connectivity and regional cerebral blood flow reveals a physiological basis for network hubs of the human brain. Proc Natl Acad Sci USA. 2013;110(5):1929–1934. 10.1073/pnas.1214900110 PMC3562840 23319644

[53] Banks SJ, Zhuang X, Bayram E, Bird C, Cordes D, Caldwell JZ, Cummings JL, Alzheimer’s Disease Neuroimaging Initiative. Default mode network lateralization and memory in healthy aging and Alzheimer’s disease. J Alzheimers Dis. 2018;66(3):1223–1234. 10.3233/JAD-180541 PMC6294587 30412488

[54] Zhang B, Pu J, Hu T, Zeng J, Zhang H, Chen Z, Ji X, Yue S, Li LZ, Li T. AI-powered nonlinear optical imaging reveals protein spatial homogenization as an indicator of impaired bone quality in type 2 diabetes. Opto-Electron Adv. 2026;9(5):250312.

[55] Zhang B, Chen Z, Li W, Xu L, Yang S, Wang F, Yan K, Wei X, Li T. Frequency-specific transcranial photobiomodulation elicits complementary glial mechanisms for neurovascular protection and amyloid clearance in Alzheimer disease. *Cyborg Bionic Syst*. 2026;7:0551. 10.34133/cbsystems.0551 PMC13287457 42344710

[56] Edde M, Leroux G, Altena E, Chanraud S. Functional brain connectivity changes across the human life span: From fetal development to old age. J Neurosci Res. 2021;99(1):236–262. 10.1002/jnr.24669 32557768

[57] Liu T, Wang L, Suo D, Zhang J, Wang K, Wang J, Chen D, Yan T. Resting-state functional MRI of healthy adults: Temporal dynamic brain Coactivation patterns. Radiology. 2022;304(3):624–632. 10.1148/radiol.211762 35503014

[58] Chhatwal JP, Schultz AP, Johnson KA, Hedden T, Jaimes S, Benzinger TL, Jack C Jr, Ances BM, Ringman JM, Marcus DS, et al. Preferential degradation of cognitive networks differentiates Alzheimer’s disease from ageing. Brain. 2018;141(5):1486–1500. 10.1093/brain/awy053 PMC5917745 29522171

[59] Sperling RA, Laviolette PS, O’Keefe K, O’Brien J, Rentz DM, Pihlajamaki M, Marshall G, Hyman BT, Selkoe DJ, Hedden T, et al. Amyloid deposition is associated with impaired default network function in older persons without dementia. Neuron. 2009;63(2):178–188. 10.1016/j.neuron.2009.07.003 PMC2738994 19640477

[60] Sheline YI, Raichle ME, Snyder AZ, Morris JC, Head D, Wang S, Mintun MA. Amyloid plaques disrupt resting state default mode network connectivity in cognitively normal elderly. Biol Psychiatry. 2010;67(6):584–587. 10.1016/j.biopsych.2009.08.024 PMC2829379 19833321

[61] Smallwood J, Bernhardt BC, Leech R, Bzdok D, Jefferies E, Margulies DS. The default mode network in cognition: A topographical perspective. Nat Rev Neurosci. 2021;22(8):503–513. 10.1038/s41583-021-00474-4 34226715

[62] Huang AW, Barber AD. Development of lateral pulvinar resting state functional connectivity and its role in attention. Cortex. 2021;136:77–88. 10.1016/j.cortex.2020.12.004 PMC8204675 33486158

[63] Squire LR, Zola-Morgan S. The medial temporal lobe memory system. Science. 1991;253(5026):1380–1386. 10.1126/science.1896849 1896849

[64] van Strien NM, Cappaert NL, Witter MP. The anatomy of memory: An interactive overview of the parahippocampal-hippocampal network. Nat Rev Neurosci. 2009;10(4):272–282. 10.1038/nrn2614 19300446

[65] Ritchey M, Montchal ME, Yonelinas AP, Ranganath C. Delay-dependent contributions of medial temporal lobe regions to episodic memory retrieval. Elife. 2015;4: Article e05025. 10.7554/eLife.05025 PMC4337612 25584461

[66] Simons JS, Spiers HJ. Prefrontal and medial temporal lobe interactions in long-term memory. Nat Rev Neurosci. 2003;4(8):637–648. 10.1038/nrn1178 12894239

[67] Khan UA, Liu L, Provenzano FA, Berman DE, Profaci CP, Sloan R, Mayeux R, Duff KE, Small SA. Molecular drivers and cortical spread of lateral entorhinal cortex dysfunction in preclinical Alzheimer’s disease. Nat Neurosci. 2014;17(2):304–311. 10.1038/nn.3606 PMC4044925 24362760

[68] Cole MW, Reynolds JR, Power JD, Repovs G, Anticevic A, Braver TS. Multi-task connectivity reveals flexible hubs for adaptive task control. Nat Neurosci. 2013;16(9):1348–1355. 10.1038/nn.3470 PMC3758404 23892552

[69] Uddin LQ, Yeo BTT, Spreng RN. Towards a universal taxonomy of macro-scale functional human brain networks. Brain Topogr. 2019;32(6):926–942. 10.1007/s10548-019-00744-6 PMC7325607 31707621

[70] Saalmann YB, Pinsk MA, Wang L, Li X, Kastner S. The pulvinar regulates information transmission between cortical areas based on attention demands. Science. 2012;337(6095):753–756. 10.1126/science.1223082 PMC3714098 22879517

[71] Hakamata Y, Mizukami S, Komi S, Sato E, Moriguchi Y, Motomura Y, Maruo K, Izawa S, Kim Y, Hanakawa T, et al. Attentional bias modification alters intrinsic functional network of attentional control: A randomized controlled trial. J Affect Disord. 2018;238:472–481. 10.1016/j.jad.2018.06.018 29929157

[72] Yeo BT, Krienen FM, Sepulcre J, Sabuncu MR, Lashkari D, Hollinshead M, Roffman JL, Smoller JW, Zöllei L, Polimeni JR, et al. The organization of the human cerebral cortex estimated by intrinsic functional connectivity. J Neurophysiol. 2011;106(3):1125–1165. 10.1152/jn.00338.2011 PMC3174820 21653723

[73] Avelar-Pereira B, Bäckman L, Wåhlin A, Nyberg L, Salami A. Age-related differences in dynamic interactions among default mode, Frontoparietal control, and dorsal attention networks during resting-state and interference resolution. Front Aging Neurosci. 2017;9:152. 10.3389/fnagi.2017.00152 PMC5438979 28588476

[74] Spreng RN, Sepulcre J, Turner GR, Stevens WD, Schacter DL. Intrinsic architecture underlying the relations among the default, dorsal attention, and frontoparietal control networks of the human brain. J Cogn Neurosci. 2013;25(1):74–86. 10.1162/jocn_a_00281 PMC3816715 22905821

[75] Valera-Bermejo JM, De Marco M, Venneri A. Altered interplay among large-scale brain functional networks modulates multi-domain Anosognosia in early Alzheimer’s disease. Front Aging Neurosci. 2021;13: Article 781465. 10.3389/fnagi.2021.781465 PMC8851037 35185517

[76] Tondelli M, Ballotta D, Maramotti R, Carbone C, Gallingani C, MacKay C, Pagnoni G, Chiari A, Zamboni G. Resting-state networks and anosognosia in Alzheimer’s disease. Front Aging Neurosci. 2024;16:1415994. 10.3389/fnagi.2024.1415994 PMC11188402 38903902

[77] Li Y, An S, Zhou T, Su C, Zhang S, Li C, Jiang J, Mu Y, Yao N, Huang ZG, et al. Triple-network analysis of Alzheimer’s disease based on the energy landscape. Front Neurosci. 2023;17:1171549. 10.3389/fnins.2023.1171549 PMC10242117 37287802

[78] Balthazar ML, de Campos BM, Franco AR, Damasceno BP, Cendes F. Whole cortical and default mode network mean functional connectivity as potential biomarkers for mild Alzheimer’s disease. Psychiatry Res. 2014;221(1):37–42. 10.1016/j.pscychresns.2013.10.010 24268581

[79] Achard S, Salvador R, Whitcher B, Suckling J, Bullmore ED. A resilient, low-frequency, small-world human brain functional network with highly connected association cortical hubs. J Neurosci. 2006;26(1):63–72. 10.1523/JNEUROSCI.3874-05.2006 PMC6674299 16399673

[80] Bullmore E, Sporns O. The economy of brain network organization. Nat Rev Neurosci. 2012;13(5):336–349. 10.1038/nrn3214 22498897

[81] Dai Z, Yan C, Li K, Wang Z, Wang J, Cao M, Lin Q, Shu N, Xia M, Bi Y, et al. Identifying and mapping connectivity patterns of brain network hubs in Alzheimer’s disease. Cereb Cortex. 2015;25(10):3723–3742. 10.1093/cercor/bhu246 25331602

[82] Liu C, Han T, Xu Z, Liu J, Zhang M, Du J, Zhou Q, Duan Y, Li Y, Wang J, et al. Modulating gamma oscillations promotes brain connectivity to improve cognitive impairment. Cereb Cortex. 2022;32(12):2644–2656. 10.1093/cercor/bhab371 34751749

[83] Corbetta M, Shulman GL. Control of goal-directed and stimulus-driven attention in the brain. Nat Rev Neurosci. 2002;3(3):201–215. 10.1038/nrn755 11994752

[84] McKhann GM, Knopman DS, Chertkow H, Hyman BT, Jack CR Jr, Kawas CH, Klunk WE, Koroshetz WJ, Manly JJ, Mayeux R, et al. The diagnosis of dementia due to Alzheimer’s disease: Recommendations from the National Institute on Aging-Alzheimer’s Association workgroups on diagnostic guidelines for Alzheimer’s disease. Alzheimers Dement. 2011;7(3):263–269. 10.1016/j.jalz.2011.03.005 PMC3312024 21514250

[85] Jack CR, Bennett DA, Blennow K, Carrillo MC, Dunn B, Haeberlein SB, Holtzman DM, Jagust W, Jessen F, Karlawish J, et al. NIA-AA research framework: Toward a biological definition of Alzheimer’s disease. Alzheimers Dement. 2018;14(4):535–562. 10.1016/j.jalz.2018.02.018 PMC5958625 29653606

[86] Hughes CP, Berg L, Danziger WL, Coben LA, Martin RL. A new clinical scale for the staging of dementia. Br J Psychiatry. 1982;140(6):566–572. 10.1192/bjp.140.6.566 7104545

[87] Folstein MF, Folstein SE, McHugh PR. A practical method for grading the cognitive state of patients for the clinician. J Psychiatr Res. 1975;12(3):189–198. 10.1016/0022-3956(75)90026-6 1202204

[88] Nasreddine ZS, Phillips NA, Bédirian V, Charbonneau S, Whitehead V, Collin I, Cummings JL, Chertkow H. The Montreal cognitive assessment, MoCA: A brief screening tool for mild cognitive impairment. J Am Geriatr Soc. 2005;53(4):695–699. 10.1111/j.1532-5415.2005.53221.x 15817019

[89] Rosen WG, Mohs RC, Davis KL. A new rating scale for Alzheimer’s disease. Am J Psychiatry. 1984;141(11):1356–1364. 10.1176/ajp.141.11.1356 6496779

[90] Maj M, D’Elia L, Satz P, Janssen R, Zaudig M, Uchiyama C, Starace F, Galderisi S, Chervinsky A. Evaluation of two new neuropsychological tests designed to minimize cultural bias in the assessment of HIV-1 seropositive persons: A WHO study. Arch Clin Neuropsychol. 1993;8(2):123–135. 14589670

[91] Lezak MD, Howieson DB, Bigler ED. *Neuropsychological assessment*. 5th ed. New York (NY): Oxford University Press; 2012.

[92] Williams BW, Mack W, Henderson VW. Boston naming test in Alzheimer’s disease. Neuropsychologia. 1989;27(8):1073–1079. 10.1016/0028-3932(89)90186-3 2797414

[93] Wei M, Shi J, Li T, Ni J, Zhang X, Li Y, Kang S, Ma F, Xie H, Qin B, et al. Diagnostic accuracy of the Chinese version of the trail-making test for screening cognitive impairment. J Am Geriatr Soc. 2018;66(1):92–99. 10.1111/jgs.15135 29135021

[94] Cummings JL, Mega M, Gray K, Rosenberg-Thompson S, Carusi DA, Gornbein J. The neuropsychiatric inventory: Comprehensive assessment of psychopathology in dementia. Neurology. 1994;44(12):2308–2314. 10.1212/wnl.44.12.2308 7991117

[95] Song P, Li S, Hao W, Wei M, Liu J, Lin H, Hu S, Dai X, Wang J, Wang R, et al. Corticospinal excitability enhancement with simultaneous transcranial near-infrared stimulation and anodal direct current stimulation of motor cortex. Clin Neurophysiol. 2021;132(5):1018–1024. 10.1016/j.clinph.2021.01.020 33743296

[96] Fan L, Li H, Zhuo J, Zhang Y, Wang J, Chen L, Yang Z, Chu C, Xie S, Laird AR, et al. The human Brainnetome atlas: A new brain atlas based on connectional architecture. Cereb Cortex. 2016;26(8):3508–3526. 10.1093/cercor/bhw157 PMC4961028 27230218

[97] Li F, Peng W, Jiang Y, Song L, Liao Y, Yi C, Zhang L, Si Y, Zhang T, Wang F, et al. The dynamic brain networks of motor imagery: Time-varying causality analysis of scalp EEG. Int J Neural Syst. 2019;29(1):1850016. 10.1142/S0129065718500168 29793372

[98] Li F, Jiang L, Zhang Y, Huang D, Wei X, Jiang Y, Yao D, Xu P, Li H. The time-varying networks of the wrist extension in post-stroke hemiplegic patients. Cogn Neurodyn. 2022;16(4):757–766. 10.1007/s11571-021-09738-2 PMC9279526 35847531

[99] Song P, Lin H, Li S, Wang L, Liu J, Li N, Wang Y. Repetitive transcranial magnetic stimulation (rTMS) modulates time-varying electroencephalography (EEG) network in primary insomnia patients: A TMS-EEG study. Sleep Med. 2019;56:157–163. 10.1016/j.sleep.2019.01.007 30871961

[100] Song P, Lin H, Liu C, Jiang Y, Lin Y, Xue Q, Xu P, Wang Y. Transcranial magnetic stimulation to the middle frontal gyrus during attention modes induced dynamic module reconfiguration in brain networks. Front Neuroinform. 2019;13:22. 10.3389/fninf.2019.00022 PMC6456710 31001103

