# Supplementary material for: Bilateral Angular Gyrus 810-nm Transcranial Near-Infrared Stimulation Reshapes Brain Network: Ameliorate Alzheimer’s Disease Cognitive Deficits
Source: BME Front. 2026 Sep 22;7:0313. doi: 10.34133/bmef.0313 (PMC13594423; doi:10.34133/bmef.0313)
Supplement: Supplementary file 1 [file bmef.0313.f1.zip › Supplementary_Material_1.docx]

**Supplementary Material-1**

**Table S1.**

***Effect sizes of within-group comparisons across three time points in the tNIRS group (Friedman test).***

| **Measure** | **n** | **χ²** | **df** | **p-value** | **Kendall‘s W** | **Effect size** |
| --- | --- | --- | --- | --- | --- | --- |
| **MMSE** | 18 | 25.529 | 2 | <0.001 | 0.709 | Large |
| **MoCA** | 18 | 25.529 | 2 | <0.001 | 0.709 | Large |
| **ADAS-cog** | 18 | 24.269 | 2 | <0.001 | 0.674 | Large |
| **AVLT-immediate recall** | 18 | 34.111 | 2 | <0.001 | 0.948 | Large |
| **AVLT-delayed recall** | 18 | 26.310 | 2 | <0.001 | 0.731 | Large |
| **AVLT-recognition** | 18 | 13.508 | 2 | 0.001 | 0.375 | Moderate |
| **BNT** | 18 | 32.413 | 2 | <0.001 | 0.900 | Large |
| **TMT-A** | 18 | 17.333 | 2 | <0.001 | 0.481 | Moderate |
| **TMT-B** | 18 | 8.885 | 2 | 0.012 | 0.247 | Small |
| **NPI** | 18 | 18.711 | 2 | <0.001 | 0.520 | Large |
| **DST-forward** | 18 | 24.128 | 2 | <0.001 | 0.670 | Large |
| **DST-backward** | 18 | 23.551 | 2 | <0.001 | 0.654 | Large |

Abbreviations: tNIRS, transcranial near-infrared stimulation; MMSE, Mini-Mental State Examination; MoCA, Montreal Cognitive Assessment; ADAS-cog, Alzheimer’s Disease Assessment Scale-Cognitive Subscale; AVLT, Auditory Verbal Learning Test; BNT, Boston Naming Test; TMT, Trail Making Test; NPI, Neuropsychiatric Inventory; DST, Digit Span Test. Effect size interpretation: Kendall‘s W ≥ 0.50 = Large, 0.30-0.49 = Moderate, 0.10-0.29 = Small.

**Table S2.**

***Pairwise within-group comparisons in the tNIRS group (Wilcoxon signed-rank tests).***

| **Measure** | **Baseline** - **Day 15** | **Day 15** - **Day 75** | **Baseline** - **Day 75** |
| --- | --- | --- | --- |
| **MMSE** | Z = −3.782, p < 0.001, r = 0.891 | Z = −3.072, p = 0.002, r = 0.724 | Z = −2.191, p = 0.028, r = 0.516 |
| **MoCA** | Z = −3.739, p < 0.001, r = 0.881 | Z = −3.502, p < 0.001, r = 0.825 | Z = −2.338, p = 0.019, r = 0.551 |
| **ADAS-cog** | Z = −3.531, p < 0.001, r = 0.832 | Z = −2.754, p = 0.006, r = 0.649 | Z = −3.113, p = 0.002, r = 0.734 |
| **AVLT-immediate recall** | Z = −3.743, p < 0.001, r = 0.882 | Z = −3.694, p < 0.001, r = 0.871 | Z = −3.759, p < 0.001, r = 0.886 |
| **AVLT-delayed recall** | Z = −3.430, p = 0.001, r = 0.808 | Z = −3.331, p = 0.001, r = 0.785 | Z = −3.066, p = 0.002, r = 0.723 |
| **AVLT-recognition** | Z = −3.276, p = 0.001, r = 0.772 | Z = −3.168, p = 0.002, r = 0.747 | Z = −1.932, p = 0.053, r = 0.455* |
| **BNT** | Z = −3.768, p < 0.001, r = 0.888 | Z = −3.835, p < 0.001, r = 0.904 | Z = −2.332, p = 0.020, r = 0.550 |
| **TMT-A** | Z = −3.462, p = 0.001, r = 0.816 | Z = −1.241, p = 0.215, r = 0.293* | Z = −3.332, p = 0.001, r = 0.785 |
| **TMT-B** | Z = −3.045, p = 0.002, r = 0.718 | Z = −2.840, p = 0.005, r = 0.669 | Z = −1.726, p = 0.084, r = 0.407* |
| **NPI** | Z = −3.066, p = 0.002, r = 0.723 | Z = −2.141, p = 0.032, r = 0.505 | Z = −2.669, p = 0.008, r = 0.629 |
| **DST-forward** | Z = −3.626, p < 0.001, r = 0.855 | Z = −3.217, p = 0.001, r = 0.758 | Z = −1.342, p = 0.180, r = 0.316* |
| **DST-backward** | Z = −3.690, p < 0.001, r = 0.870 | Z = −3.153, p = 0.002, r = 0.743 | Z = −2.000, p = 0.046, r = 0.471 |

*Statistically non-significant (p ≥ 0.05). Effect size interpretation: r ≥ 0.50 = Large; 0.30–0.49 = Moderate; 0.10–0.29 = Small.

Data are presented as Z (p-value), [effect size r]. t0 = baseline; t1 = post-intervention (Day 15); t2 = follow-up (Day 75).

Negative Z values indicate improvement (lower scores for ADAS-cog and NPI; higher scores for all other measures).

**Table S3.**

***Between-group comparisons at each time point (Mann-Whitney U tests).***

| **Measure** | **Baseline** | **Day 15** | **Day 75** |
| --- | --- | --- | --- |
| **MMSE** | Z = −0.14, p = 0.887, r = 0.023 | Z = −3.38, p = 0.001, r = 0.563 | Z = −3.28, p = 0.001, r = 0.547 |
| **MoCA** | Z = −0.62, p = 0.536, r = 0.103 | Z = −3.55, p < 0.001, r = 0.592 | Z = −2.58, p = 0.010, r = 0.430 |
| **ADAS-cog** | Z = 1.68, p = 0.093, r = 0.280 | Z = 3.81, p < 0.001, r = 0.635 | Z = 3.75, p < 0.001, r = 0.625 |
| **AVLT-immediate recall** | Z = −0.50, p = 0.617, r = 0.083 | Z = −3.54, p < 0.001, r = 0.590 | Z = −2.81, p = 0.005, r = 0.468 |
| **AVLT-delayed recall** | Z = 0.77, p = 0.441, r = 0.128 | Z = −3.35, p = 0.001, r = 0.558 | Z = −2.69, p = 0.007, r = 0.448 |
| **AVLT-recognition** | Z = −1.64, p = 0.101, r = 0.273 | Z = −3.42, p = 0.001, r = 0.570 | Z = −2.52, p = 0.012, r = 0.420 |
| **BNT** | Z = −0.17, p = 0.865, r = 0.028 | Z = −3.72, p < 0.001, r = 0.620 | Z = −2.64, p = 0.008, r = 0.440 |
| **TMT-A** | Z = 1.13, p = 0.258, r = 0.188 | Z = 2.78, p = 0.005, r = 0.463 | Z = 2.60, p = 0.009, r = 0.433 |
| **TMT-B** | Z = −0.01, p = 0.992, r = 0.002 | Z = −0.42, p = 0.675, r = 0.070* | Z = −0.58, p = 0.562, r = 0.097* |
| **NPI** | Z = −0.39, p = 0.697, r = 0.065 | Z = 2.38, p = 0.017, r = 0.397 | Z = −1.79, p = 0.073, r = 0.298* |
| **DST-forward** | Z = −0.13, p = 0.897, r = 0.022 | Z = −3.48, p = 0.001, r = 0.580 | Z = −1.85, p = 0.064, r = 0.308* |
| **DST-backward** | Z = −0.53, p = 0.596, r = 0.088 | Z = −3.39, p = 0.001, r = 0.565 | Z = −1.22, p = 0.222, r = 0.203* |

*Statistically non-significant (p ≥ 0.05). Effect size interpretation: r ≥ 0.50 = Large; 0.30–0.49 = Moderate; 0.10–0.29 = Small.

Positive Z values indicate better performance in the tNIRS group (except for NPI, where positive Z indicates lower — i.e., improved — scores in the tNIRS group).

**Table S4.**

***Summary of effect sizes (r) for within-group comparisons in the tNIRS group.***

| **Measure** | **Baseline** - **Day 15** | **Day 15** - **Day 75** | **Baseline** - **Day 75** |
| --- | --- | --- | --- |
| **MMSE** | 0.891 (Large) | 0.724 (Large) | 0.516 (Large) |
| **MoCA** | 0.881 (Large) | 0.825 (Large) | 0.551 (Large) |
| **ADAS-cog** | 0.832 (Large) | 0.649 (Large) | 0.734 (Large) |
| **AVLT-immediate recall** | 0.882 (Large) | 0.871 (Large) | 0.886 (Large) |
| **AVLT-delayed recall** | 0.808 (Large) | 0.785 (Large) | 0.723 (Large) |
| **AVLT-recognition** | 0.772 (Large) | 0.747 (Large) | 0.455 (Moderate)* |
| **BNT** | 0.888 (Large) | 0.904 (Large) | 0.550 (Large) |
| **TMT-A** | 0.816 (Large) | 0.293 (Small)* | 0.785 (Large) |
| **TMT-B** | 0.718 (Large) | 0.669 (Large) | 0.407 (Moderate)* |
| **NPI** | 0.723 (Large) | 0.505 (Large) | 0.629 (Large) |
| **DST-forward** | 0.855 (Large) | 0.758 (Large) | 0.316 (Moderate)* |
| **DST-backward** | 0.870 (Large) | 0.743 (Large) | 0.471 (Moderate) |

*Statistically non-significant (p ≥ 0.05). Effect size interpretation: r ≥ 0.50 = Large; 0.30–0.49 = Moderate; 0.10–0.29 = Small.

**Table S5.**

***Summary of effect sizes (r) for between-group comparisons (tNIRS vs. sham).***

| **Measure** | **Baseline** | **Post-intervention (Day 15)** | **Follow-up (Day 75)** |
| --- | --- | --- | --- |
| **MMSE** | 0.023 (Small) | 0.563 (Large) | 0.547 (Large) |
| **MoCA** | 0.103 (Small) | 0.592 (Large) | 0.430 (Moderate) |
| **ADAS-cog** | 0.280 (Small) | 0.635 (Large) | 0.625 (Large) |
| **AVLT-immediate recall** | 0.083 (Small) | 0.590 (Large) | 0.468 (Moderate) |
| **AVLT-delayed recall** | 0.128 (Small) | 0.558 (Large) | 0.448 (Moderate) |
| **AVLT-recognition** | 0.273 (Small) | 0.570 (Large) | 0.420 (Moderate) |
| **BNT** | 0.028 (Small) | 0.620 (Large) | 0.440 (Moderate) |
| **TMT-A** | 0.188 (Small) | 0.463 (Moderate) | 0.433 (Moderate) |
| **TMT-B** | 0.002 (Small) | 0.070 (Small)* | 0.097 (Small)* |
| **NPI** | 0.065 (Small) | 0.397 (Moderate) | 0.298 (Small)* |
| **DST-forward** | 0.022 (Small) | 0.580 (Large) | 0.308 (Moderate)* |
| **DST-backward** | 0.088 (Small) | 0.565 (Large) | 0.203 (Small)* |

*Statistically non-significant (p ≥ 0.05). Effect size interpretation: r ≥ 0.50 = Large; 0.30–0.49 = Moderate; 0.10–0.29 = Small.

**Abbreviations:**

AVLT = Auditory Verbal Learning Test; BNT = Boston Naming Test; TMT = Trail Making Test; NPI = Neuropsychiatric Inventory; DST = Digit Span Test.

MMSE = Mini-Mental State Examination; MoCA = Montreal Cognitive Assessment; ADAS-cog = Alzheimer’s Disease Assessment Scale — Cognitive subscale.

All p-values are two-tailed. Effect sizes (r) were calculated as |Z|/√n. Statistical significance threshold: α = 0.05.

**Table S6.**

***Exploratory subgroup analysis comparing pre-to-post intervention changes (Δ) between patients with and without concomitant AD medications in the tNIRS group.***

| **Measure** | **With medication**  **(n = 8)** | **Without medication**  **(n = 10)** | **p-value*** |
| --- | --- | --- | --- |
| **MMSE** | 2.88 ± 1.13 | 3.40 ± 1.35 | 0.535 |
| **MoCA** | 4.75 ± 2.25 | 3.80 ± 3.12 | 0.720 |
| **ADAS-cog** | −5.13 ± 4.02 | −6.80 ± 2.66 | 0.200 |
| **AVLT-immediate recall** | 5.25 ± 2.92 | 4.80 ± 2.20 | 0.760 |
| **BNT** | 2.25 ± 1.67 | 2.30 ± 1.06 | 0.944 |
| **NPI** | −2.63 ± 3.20 | −3.30 ± 4.08 | 0.682 |

**Abbreviations:**

AD, Alzheimer’s disease; tNIRS, transcranial near-infrared stimulation; Δ, post-intervention (Day 15) minus baseline; MMSE, Mini-Mental State Examination; MoCA, Montreal Cognitive Assessment; ADAS-cog, Alzheimer’s Disease Assessment Scale—Cognitive Subscale; AVLT, Auditory Verbal Learning Test; BNT, Boston Naming Test; NPI, Neuropsychiatric Inventory.

**Notes:**

Patients on stable doses of anti-dementia medications were classified into the “with medication” group.

For ADAS-cog and NPI, negative Δ values indicate improvement.

Data were analyzed using the Mann-Whitney U test.

All p-values are two-tailed.

No significant between-subgroup differences were observed for any measure (all p > 0.05).

**Table S7.**

***Proportion of patients achieving MCID thresholds in the tNIRS group.***

| **Measure** | **MCID threshold** | **Responders (n/N)** | **Response rate (%)** |
| --- | --- | --- | --- |
| **MMSE** | ≥ +2 points | 16/18 | 88.9 |
| **ADAS-Cog** | ≥ −3 points | 16/18 | 88.9 |
